# Polygenic Risk Score Effectively Predicts Depression Onset in Alzheimer’s Disease Based on Major Depressive Disorder Risk Variants

**DOI:** 10.3389/fnins.2022.827447

**Published:** 2022-03-08

**Authors:** Suraj Upadhya, Hongliang Liu, Sheng Luo, Michael W. Lutz, Ornit Chiba-Falek

**Affiliations:** ^1^Division of Translational Brain Sciences, Department of Neurology, Duke University Medical Center, Durham, NC, United States; ^2^Department of Biostatistics and Bioinformatics, Duke University Medical Center, Durham, NC, United States; ^3^Department of Population Health Sciences, Duke University School of Medicine, Durham, NC, United States; ^4^Center for Genomic and Computational Biology, Duke University Medical Center, Durham, NC, United States

**Keywords:** polygenic risk scores, depression, late-onset Alzheimer’s disease, neuropsychiatric symptoms, heterogeneity, major depressive disorder

## Abstract

**Introduction:**

Depression is a common, though heterogenous, comorbidity in late-onset Alzheimer’s Disease (LOAD) patients. In addition, individuals with depression are at greater risk to develop LOAD. In previous work, we demonstrated shared genetic etiology between depression and LOAD. Collectively, these previous studies suggested interactions between depression and LOAD. However, the underpinning genetic heterogeneity of depression co-occurrence with LOAD, and the various genetic etiologies predisposing depression in LOAD, are largely unknown.

**Methods:**

Major Depressive Disorder (MDD) genome-wide association study (GWAS) summary statistics were used to create polygenic risk scores (PRS). The Religious Orders Society and Rush Memory and Aging Project (ROSMAP, *n* = 1,708) and National Alzheimer’s Coordinating Center (NACC, *n* = 10,256) datasets served as discovery and validation cohorts, respectively, to assess the PRS performance in predicting depression onset in LOAD patients.

**Results:**

The PRS showed marginal results in standalone models for predicting depression onset in both ROSMAP (AUC = 0.540) and NACC (AUC = 0.527). Full models, with baseline age, sex, education, and *APOEε4* allele count, showed improved prediction of depression onset (ROSMAP AUC: 0.606, NACC AUC: 0.581). In time-to-event analysis, standalone PRS models showed significant effects in ROSMAP (*P* = 0.0051), but not in NACC cohort. Full models showed significant performance in predicting depression in LOAD for both datasets (*P* < 0.001 for all).

**Conclusion:**

This study provided new insights into the genetic factors contributing to depression onset in LOAD and advanced our knowledge of the genetics underlying the heterogeneity of depression in LOAD. The developed PRS accurately predicted LOAD patients with depressive symptoms, thus, has clinical implications including, diagnosis of LOAD patients at high-risk to develop depression for early anti-depressant treatment.

## Introduction

Neuropsychiatric symptoms (NPS) are common in Late-onset Alzheimer’s Disease (LOAD), characterized by heterogeneity with highly variable onset duration and severity. Amongst LOAD with comorbid NPS, depression and anxiety are the most prevalent (Lyketsos et al., 2011; Lyketsos, 2015; Zhao et al., 2016; Hallikainen et al., 2018; Banning et al., 2021). Furthermore, individuals with depression are at greater risk to develop LOAD, suggesting that treating depression may delay LOAD (Lyketsos et al., 2011; Lutz et al., 2020). In addition, distinct trajectories of increasing risk of depression were associated with LOAD pathology such as, lower cerebrospinal fluid (CSF) Aβ_42_ and higher CSF total and phosphorylated tau, highlighting the heterogeneity of depression within LOAD (Banning et al., 2021). Interestingly, we previously identified shared genetic etiology between LOAD and major depressive disorder (MDD) (Lutz et al., 2020). Moreover, several groups have investigated the mechanisms underlying the genetic interaction between depression and dementia (Chi et al., 2014; Brzezińska et al., 2020). Their findings implicated genes regulating the inflammatory and immune responses, and genes in the endocytosis pathway in both depression and LOAD (Chi et al., 2014; Lutz et al., 2020). Noteworthy, *APOEε*4, known as the strongest genetic risk factor for LOAD, has been associated with increased risk for depression in several studies (Geda et al., 2006; Chi et al., 2014; Feng et al., 2015; Wang et al., 2019). Collectively, this evidence lends support for inter-relationships between LOAD and depression disorders (Lutz et al., 2020).

Polygenic risk scores (PRS) offer a method to explore such relationships that may exist between LOAD and depression. The current LOAD polygenic risk scores (PRS) landscape focuses on predicting LOAD diagnosis (Escott-Price et al., 2015, 2017, 2019; Tasaki et al., 2018; Leonenko et al., 2019; Altmann et al., 2020), with a few studies applying pathway and functional analysis to the selection of SNPs for PRS calculation (Darst et al., 2017; Tesi et al., 2020). LOAD PRS have been tested to predict mild cognitive impairment (MCI) to LOAD progression (Leonenko et al., 2019; Altmann et al., 2020; Daunt et al., 2021). Additionally, studies have tested PRS association with LOAD phenotypes in CSF biomarkers (Darst et al., 2017; Tasaki et al., 2018; Leonenko et al., 2019; Altmann et al., 2020; Tesi et al., 2020; Daunt et al., 2021; Zettergren et al., 2021) and motor-function impairment (Tasaki et al., 2019). Other than associations with biomarker data, the effectiveness of PRS to predict LOAD heterogenous endophenotypes especially comorbid NPS, including depression, has yet to be thoroughly examined.

In this study we generated and tested the effectiveness of PRS to predict depression risk and onset time course in LOAD patients. We created a novel PRS based on MDD genome-wide association study (GWAS) summary statistics and examined its utility in predicting the risk to develop depression symptoms in LOAD patients using two well-characterized LOAD cohorts from the Religious Orders Study and Rush Memory and Aging Project (ROSMAP) (Bennett et al., 2012a,b, 2018) and National Alzheimer’s Coordinating Center (NACC) (Beekly et al., 2004) projects.

## Materials and Methods

### Study Cohorts

**FIGURE 1:**
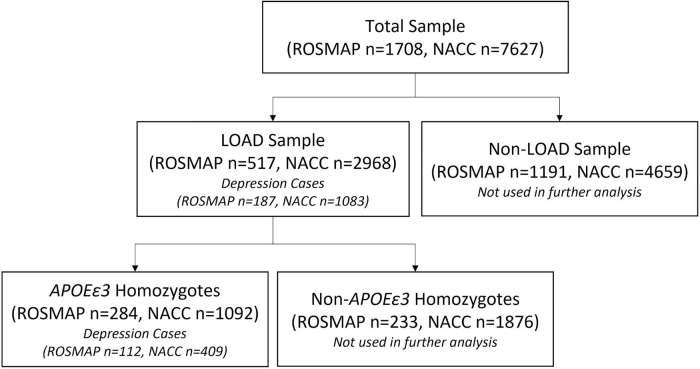
Sample selection flowchart. The total samples from both ROSMAP and NACC datasets were divided into LOAD and non-LOAD groups, where the non-LOAD group was not studied. Depression case and controls were identified in the LOAD sample of both datasets. *APOEε3* homozygotes were then selected from the LOAD sample to account for potential confounding by the *APOEε4* allele.

Two cohorts were used to evaluate the performance of the PRS in predicting risk of depression onset: ROSMAP and NACC, whereas ROSMAP cohort was used for discovery and NACC dataset was used for validation. We used only the samples that had available genetic data and information on depression phenotypes. All samples were LOAD patients. Cases were defined as LOAD with depression symptoms, and controls were LOAD individuals who did not experience depression (Figure 1). To further control for *APOE* as a cofounding factor, we repeated the analyses using sub-cohorts stratified into *APOEε3* homozygotes (Figure 1). Of note, the ROSMAP sample is also included in the NACC data. Table 1 summarized the descriptive statistics for the ROSMAP and NACC samples used in this study.

**TABLE 1:** Sample demographics.

	ROSMAP (*n* = 1,708)		NACC (*n* = 7,627)	
Sample	Full LOAD sample	*APOEε3* Homozygote sample	Full LOAD sample	*APOEε3* Homozygote sample
Subjects	*n* = 517	*n* = 284	*n* = 2,968	*n* = 1,092
Female %	68.0%	70.4%	52.1%	50.3%
Mean education in years (*SD*)	16.2 (3.7)	16.2 (3.8)	15.6 (6.9)	15.7 (7.4)
Mean baseline Age (*SD*)	81.5 (6.7)	81.8 (6.7)	76.3 (9.1)	78.0 (9.9)
Race, White %	99.8%	100%	99.7%	100%
*APOEe4* count				
0	333		1,226	
1	168	NA	1,355	NA
2	15		376	
Depression				
Cases	187	112	1,083	409
Controls	330	172	1,885	683

*LOAD, Late-onset Alzheimer’s Disease; ROSMAP, Relgious Orders Study and Rush Memory and Aging Project; NACC, National Alzheimer’s Coordinating Center; APOE, Apolipoprotein E; SD, Standard Deviation.*

#### Rush Memory and Aging Project

The discovery sample was derived from two ongoing cohort studies, the Religious Orders Study (ROS) and Rush Memory and Aging Projects (MAP) (Bennett et al., 2012a,b, 2018). ROS began recruiting nuns and brothers from across the United States in 1994, while MAP started recruiting individuals from northeastern Illinois in 1997. Both studies were conducted by the same team of investigators (Bennett et al., 2012a,b, 2018). Thus, the studies used similar data collection procedures and shared a common set of examinations, allowing for a combined analysis. Study participants were free of known dementia at enrollment, underwent annual clinical and neuropsychological evaluations, and agreed to brain donation at the time of death. The studies were approved by the Institutional Review Board of Rush University Medical Center. Written informed consent was acquired from each participant. LOAD cases were defined using the final consensus cognitive diagnosis variable, where an Alzheimer’s disease diagnosis with or without another cause of cognitive impairment (cogdx = 4 or 5) was considered a LOAD case. LOAD cases comprised the sample for further analysis, specifically prediction of risk and onset of depression. The variable r_depres (Bennett et al., 2004) indicated MDD, or depression, diagnosis by a physician. The depression criteria from the Diagnostic and Statistical Manual of Mental Disorders, 3rd Edition, Revised (DSM-III-R) (American Psychiatric Association [APA], 1987), a clinical interview with the patient, and patient’s responses to questions from the Diagnostic Interview Schedule were employed by physicians to diagnose clinical depression, with final endpoints of highly probably, probable, possible, and not possible clinical depression. A diagnosis of highly probable, probable, or possible depression (r_depres = 1, 2, or 3) in any study visit was deemed a depression case. Thus, one instance of depression in the study duration was considered a depression case. The classification of the cohort and number of subjects in each category (LOAD with depression and LOAD only) are described in a flowchart (Figure 1). Other variables included were age at baseline (age_bl), sex (msex), years of education (educ), financial_need, and apoe_genotype. The educ variable represents years of education (Bennett et al., 2005), and the financial_need variable estimates financial need during childhood (Wilson et al., 2006). This variable is only available in the MAP data. The inclusion of this variable was based on the hypothesis that chronic stress due to financial need, especially during the critical period of childhood, may have psychosocial effects later in life (Wilson et al., 2006). The apoe_genotype variable specifies the subject’s *APOE* genotype (Yu et al., 2017); this variable was then converted to another variable to count the number of *APOEε4* alleles (0, 1, 2).

Overall, 517 LOAD cases from the ROSMAP cohort (total *n* = 1,708) were used in our study, out of which there were 187 depression cases and 330 controls (i.e., only LOAD) (Figure 1). The sample consisted of 68% female, with an average age at baseline of 81.5 (*SD* = 6.7) and years of education of 16.2 (*SD* = 3.7) (Table 1). A total of 284 entire LOAD cases were *APOEε3* homozygote, with 112 cases (LOAD comorbid depression) and 172 controls (LOAD only) (Figure 1). 70.4% of which were females and average baseline age of 81.8 (*SD* = 6.7) (Table 1).

#### National Alzheimer’s Coordinating Center

The validation sample used was obtained from the National Alzheimer’s Coordinating Center (NACC) (Beekly et al., 2004). The NACC is composed of 29 Alzheimer’s Disease Research Centers (ADRC) located throughout North America. The data collection and management vary between centers, with each center enrolling based on specific research interests. Some ADRCs require subjects to agree to autopsies. Written informed consent was acquired from each subject. The primary diagnosis variable (dx) was used to select LOAD cases, with dx = 050 corresponding to Alzheimer’s Disease. The variable DEP was employed to select depression cases, with a value of 1 indicating a depression diagnosis within the last 2 years and value of 0 noting no depression diagnosis. These values result from a clinical diagnosis of depression using the Geriatric Depression Scale (GDS) (Sheikh and Yesavage, 1986). Cases were defined as participants with a diagnosis for depression at least once within the study duration, while controls were those that did not have a depression diagnosis. As with ROSMAP, any instance of depression throughout the study course was marked as a depression case. The classification of the cohort and number of subjects in each category (LOAD with depression and LOAD only) are described in a flowchart (Figure 1). Other variables included were age at baseline (NACCAGEB), sex, years of education (EDUC), and *APOE* genotype.

Overall, 2,968 LOAD cases from the entire NACC data (7,627) were used in our study. Out of which were 1,083 depression cases and 1,885 controls (i.e., only LOAD) (Figure 1). The sample consisted of 52.1% female, with an average baseline age of 76.3 (*SD* = 9.1) and years of education of 15.6 (*SD* = 6.9) (Table 1). A total of 1,092 NACC LOAD subjects were *APOEε*3 homozygotes, with 409 cases and 683 controls (Figure 1), 50.4% of which were females, and average baseline age was 78.0 (*SD* = 9.9) and years of education of 15.7 (*SD* = 7.4) (Table 1).

### Genome-Wide Association Study Data for Polygenic Risk Scores Construction

The MDD GWAS (PGC-MDD2) conducted by Wray et al. (2018) included summary statistics of *P*-values, odds ratios, standard errors, reference and alternate alleles, imputation quality score (INFO), and direction of effect from the Psychiatric Genomics Consortium. Data for 6 cohorts described by Wray et al. (2018) were acquired (PGC29, deCODE, Generation Scotland, GERA, iPSYCH, and UK Biobank) and used in this study. These results included genotyped and imputed data on 13,554,489 SNPs from 59,851 MDD cases and 113,154 controls. Primary manuscripts for the MDD GWAS (Wray et al., 2018) further describe details regarding genotyping procedure, quality control, and GWAS analysis. Constraining to SNPs with high quality imputation scores (INFO > 0.9) lead to 8,209,158 SNPs remaining. All resulting SNPs passed quality control metrics, as described by Wray et al. (2018). All genomic coordinates are based on NCBI Build 37/USCS hg19.

### Genotype Data for Polygenic Risk Scores Construction

Genotype data from 1,708 subjects in ROSMAP and 10,256 subjects in NACC were then retrieved for the target samples used for testing the PRS. For NACC, genotype data from Alzheimer’s Disease Centers 1--7 were downloaded. For both datasets, imputation was performed with minimac4 on the Michigan imputation server.^1^ For the imputation reference panel, the HRC panel (Version r1.1 2016) was used. This panel is composed of 64,940 haplotypes of mainly European ancestry. High quality SNPs were used for imputation, using the following parameters: MAF > 0.01; call rate > 95%, Hardy-Weinberg equilibrium test *P* > 10^–6^; allele frequency difference ≤ 0.20 between the sample data and the reference panel. PLINK 1.9/2 (Purcell et al., 2007) was used to process the genotype data.

### Polygenic Risk Scores Calculation

Two formulas were used to calculate PRS. Formula 1 describes the method of calculating PRS by multiplying beta values (β) by the number of effect alleles (*X*) then summing these values, which will be referenced as PRS. Formula 2 utilizes the risk allele (*G*), or the allele with the positive beta value, which will be referenced as risk-increasing PRS (Bellenguez et al., 2020). The number of risk alleles is multiplied by its respective beta value. This term is then multiplied by the total number of SNPs (*T*) divided by the sum of all the beta values. This term allows for the risk-increasing PRS to represent the average of risk alleles, providing an interpretable result in terms of risk allele (Chouraki et al., 2016; Bellenguez et al., 2020).


(1) ∑β*X

(2) $$\sum \beta *G*\frac{T}{\sum \beta }$$

The *APOE* region, defined as the ± 300 Kb around the *APOE* epsilon coding SNPs (chr19:45,111,942–45,711,941), was not included in the PRS calculation. PRSice-2 (Choi and O’Reilly, 2019) was used to produce the PRS. SNPs were selected from the MDD GWAS data using multiple *p*-value thresholds (0.5, 0.4, 0.3, 0.2, 0.1, 0.05, 0.01, 0.005, 0.001, 0.0001, 10^–5^, 10^–6^, 10^–7^, 10^–8^). Then, the genotyped data of both ROSMAP and NACC was scanned to select SNPs from the MDD GWAS data at respective *p*-value thresholds. Since all SNP genotypes were not present in both datasets, dosages for missing genotypes were set to zero, with the assumption that most of the population have at least one copy of the major allele, which is best approximated with a score of zero. ROSMAP had 5,543,088 SNPs found in the MDD GWAS data. NACC contained 3,633,901 SNPs matching selected SNPs from the MDD GWAS data. Clumping was done on the resultant SNPs to account for linkage disequilibrium (LD Parameters: *R*^2^> 0.1, *P* = 1.0, window = 250 kb). The total number of variants after clumping for ROSMAP and NACC were 315,079 and 243,437, respectively. Supplementary Table 1 details the total number of SNPs used for each *p*-value threshold and dataset.

### Statistical Analysis

Logistic regression and Receiver Operating Characteristic (ROC) curves were calculated to assess the performance of the PRS to predict depression within the LOAD only samples. These analyses were completed for all *p*-value thresholds to determine the optimum threshold for prediction, which then was then utilized in subsequent analyses. For ROSMAP, performance was assessed of a statistical prediction model that included the covariates *APOEε4* allele count, childhood financial need, sex, and the 0.005 *p*-value SNP selection threshold PRS. This was subsequently replicated in NACC, where a similar prediction model was created that included covariates education, *APOEε4* allele count, sex, and 0.005 *p*-value SNP selection threshold PRS. Prediction models excluding PRS were constructed in both ROSMAP and NACC and compared with respective models including PRS using the DeLong test (DeLong et al., 1988). Additionally, time-to-event analysis was conducted, with left-truncated (age at entry) and right censored (age at depression onset or age at last visit) data (Altmann et al., 2020). Age at depression onset was defined as the age at first depression diagnosis. Subsequently, principal component analysis (PCA) was conducted on both datasets to account for population structure, with principal components (PC) 1, 2, and 3 sufficiently accounting for the majority of the variance (Supplementary Figures 1, 2). Models from both datasets were subsequently adjusted for population structure using PC1-3. Statistical analysis was completed in JMP Pro 15 (JMP^®^, 2021) and the DeLong tests were run in the MedCalc application (MedCalc^®^, 2021).

## Results

### Polygenic Risk Scores Selection and Distribution

We created 14 PRS, for each dataset, using multiple *p*-value thresholds (hereafter *P*_Threshold_). SNPs were selected according to each *p*-value threshold (SNPs counts by *P*_Threshold_ are summarized in Supplementary Table 1). Logistic regression plots of PRS and depression phenotype were then used to select the optimal *P*_Threshold_ in ROSMAP, with the same *P*_Threshold_ used subsequently in NACC for validation. In ROSMAP, the logistic regression analysis with a *P*_Threshold_ of 0.005 resulted in the greatest effect size for the PRS term (beta = 0.153, *P* = 0.089; Supplementary Table 2). Thus, PRS generated with SNPs selected for *P*_Threshold_ of 0.005 were then employed in further evaluations in ROSMAP and validation in NACC. In NACC, the logistic regression analysis improved, with the *P*_Threshold_ of 0.005 meeting significance (beta = 0.092, *P* = 0.0149; Supplementary Table 3). Additional logistic regression results for other *P*_Threshold_ in NACC can be found in Supplementary Table 3, where these results were not considered in SNP selection.

**FIGURE 2:**
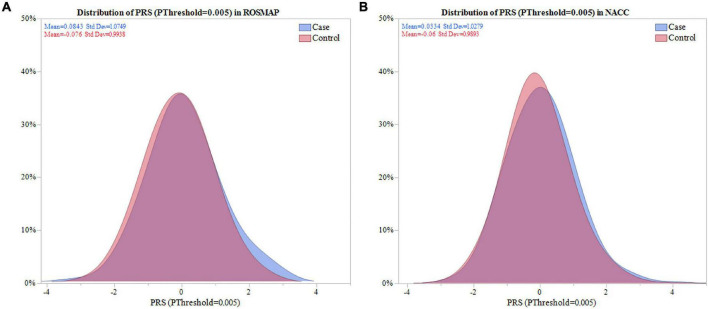
Distribution of PRS (*P*_Threshold_ = 0.005) in ROSMAP and NACC. Distribution of the PRS was compared between cases and controls in ROSMAP and NACC. **(A)** For ROSMAP, the mean of the PRS (*P*_Threshold_ = 0.005) in cases and controls was 0.084 and -0.076, respectivley. Signficance difference was found using a two sample one sided *t*-test (*P* = 0.048). **(B)** For NACC, the mean of the PRS (*P*_Threshold_ = 0.005) in cases and controls was 0.033 and -0.060, respectively. There was a significant difference amongst the means using a two sample one sided *t*-test (*P* = 0.008).

Next, the distribution of the PRS calculated with SNPs selected for *P*_Threshold_ = 0.005, hereafter PRS (*P*_Threshold_ = 0.005), was evaluated. The PRS (*P*_Threshold_ = 0.005) distribution demonstrated that depression cases in ROSMAP had a significantly higher mean PRS compared to controls (0.084 vs. -0.076, *P* = 0.048; Figure 2A). This was replicated in NACC (0.033 vs. -0.60, *P* = 0.008; Figure 2B).

### Prediction of Onset of Depression in Late-Onset Alzheimer’s Disease

The PRS distribution and logistic regression analysis test the ability of the PRS and covariates to classify cases and controls. Further evaluations were done in ROSMAP and validated in NACC to assess the predictive ability using standalone PRS models and full prediction models.

#### Rush Memory and Aging Project

**FIGURE 3:**
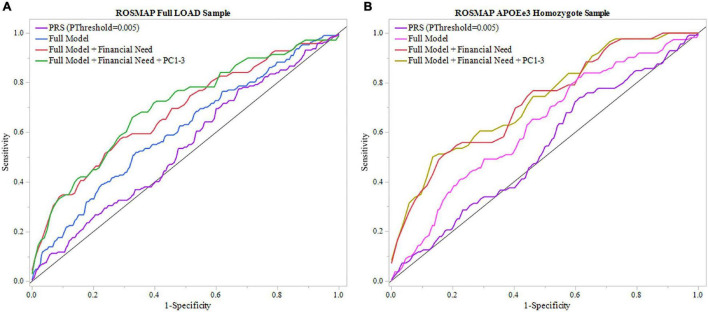
ROC analysis of PRS and full model in ROSMAP. Receiver operating characteristic (ROC) curves were used to assess diagnostic ability, with the Area Under the Curve (AUC) providing a quantitative measure. The models illustrated are PRS (*P*_Threshold_ = 0.005) alone, the full model consisting of PRS (*P*_Threshold_ = 0.005), age, sex, education, *APOEε4* allele count, full model with childhood financial need (Full Model + Financial Need), and full model with financial need and principal components 1–3 (Full Model + Financial Need + PC1-3). **(A)** Models using the full LOAD ROSMAP sample. Full model including financial need had superior results (AUC = 0.680) than the full model (AUC = 0.606) and PRS (*P*_Threshold_ = 0.005) alone (AUC = 0.540). **(B)** Similar analyses were repeated in an *APOEε3* homozygote sample to assess model performance independent of *APOEε4*. Both full model with financial need had greater results (AUC = 0.721) and full model (AUC = 0.624) had greater results than in the full LOAD sample. The PRS (*P*_Threshold_ = 0.005) alone saw a slight decline in perfromance (AUC = 0.535) compared to the full LOAD sample.

A model using only the PRS (*P*_Threshold_ = 0.005) resulted in an AUC of 0.540 (Table 2). We applied the full model, which, in addition to the PRS (*P*_Threshold_ = 0.005), included baseline age, sex, years of education, and *APOEε4* allele count (Supplementary Table 5). The model resulted in an AUC of 0.606 and was improved to an AUC of 0.680 with the inclusion of childhood financial need as an additional variable (Figure 3A and Table 2). Noteworthy, education had a significant effect in the full model (beta = –0.134, *P* = 0.033), while the PRS (*P*_Threshold_ = 0.005) had a marginal contribution (beta = 0.249, *P* = 0.126) (Supplementary Table 5). Both models, with and without childhood financial need, were then compared to the respective model without PRS (*P*_Threshold_ = 0.005) (Supplementary Table 4) to assess the increase in model performance attributed to the addition of PRS (*P*_Threshold_ = 0.005). In both models, there was no significant increase in model performance when comparing AUCs (With, without childhood financial need: *P* = 0.377, *P* = 0.774; Supplementary Table 8). Upon adjusting for population structure, both full models using the PCs, with and without financial need, improved (AUC = 0.700, AUC = 0.629; Table 2 and Supplementary Table 10). The PRS (*P*_Threshold_ = 0.005) had a similar marginal contribution (beta = 0.250, *P* = 0.129), with no significant addition to model performance when compared to model without PRS as assessed using DeLong test (With, without childhood financial need: *P* = 0.522, *P* = 0.854; Supplementary Tables 9, 13).

**TABLE 2:** Assessing PRS ability to predict risk of depression onset.

	ROSMAP		NACC	
Sample	Full LOAD sample	*APOEε3* Homozygote sample	Full LOAD sample	*APOEε3* Homozygote sample
Case-control (*p*-value)	0.088	0.277	0.015*	0.649
Full model (AUC)	0.606*^a^*	0.624*^b^*	0.581	0.587
Full model + PC1-3 (AUC)	0.629*^c^*	0.648*^d^*	0.591	0.612
PRS (AUC)	0.540	0.535	0.527	0.507

*Full Model included covariates of PRS, APOEε4 allele count, sex, baseline age, and education. Logistic regression analyses and receiver operating characteristics (ROC) curves were created to assess PRS ability to predict risk of depression onset.*

*^a^Improved to 0.680 with addition of childhood financial need.*

*^b^Improved to 0.721 with addition of childhood financial need.*

*^c^Improved to 0.700 with addition of childhood financial need.*

*^d^Improved to 0.728 with addition of childhood financial need.*

*Statistical signifiance met.

*LOAD, Late-onset Alzheimer’s Disease; ROSMAP, Relgious Orders Study and Rush Memory and Aging Project; NACC, National Alzheimer’s Coordinating Center; APOE, Apolipoprotein E; SNP, Single Nucleotide Polymorphism; AUC, Area Under the Curve; PRS, Polygenic Risk Score; PC, Principal Component.*

We repeated the analyses in the subgroup stratified for *APOEε3* homozygotes to exclude a potential confounding effect of *APOEε4* on the PRS. In this subgroup, the PRS (*P*_Threshold_ = 0.005) showed an AUC of 0.535, with an improved AUC of 0.624 in the full model, which was further improved to an AUC of 0.721 with the addition of childhood financial need (Figure 3B, Table 2, and Supplementary Table 7). Baseline age (beta = –0.115, *P* = 0.014) and childhood financial need (beta = 0.595, *P* = 0.0097) had significant effects in the full model, but the PRS (*P*_Threshold_ = 0.005) did not reach significance (beta = 0.349, *P* = 0.138) (Supplementary Table 7). Using the DeLong test to compare both models, with and without childhood financial need, to their respective models excluding PRS (*P*_Threshold_ = 0.005) (Supplementary Table 6) resulted in no significant increase in the AUC, or model performance, with the addition of PRS (With, without childhood financial need: *P* = 0.237, *P* = 0.377; Supplementary Table 8). Both full models improved when adjusting for population structure using PC1-3 (with, without childhood financial need: AUC = 0.728, AUC = 0.648; Table 2 and Supplementary Table 12). The PRS (*P*_Threshold_ = 0.005) did not have a significant contribution in the full model (beta = 0.385, *P* = 0.110), which was further supported by the DeLong test when comparing with the model excluding PRS (With, without childhood financial need: *P* = 0.361, *P* = 0.200; Supplementary Tables 11, 13). These results suggested that other factors have a greater contribution to prediction performance, while the PRS had a moderate contribution.

#### National Alzheimer’s Coordinating Center

**FIGURE 4:**
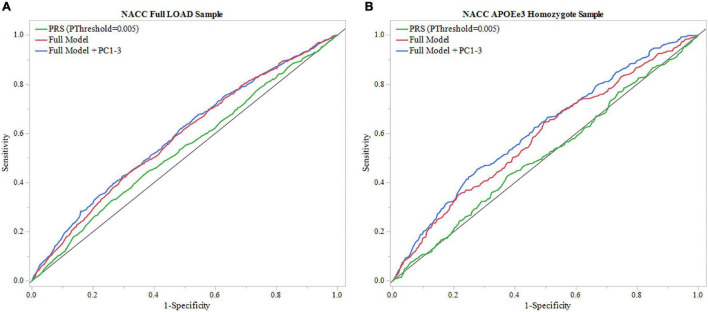
ROC analysis of PRS and full model in NACC. Receiver operating characteristic (ROC) curves were used to assess diagnostic ability, with the Area Under the Curve (AUC) providing a quantitative measure. The models illustrated are PRS (*P*_Threshold_ = 0.005) alone and the full model consisting of PRS (*P*_Threshold_ = 0.005), age, sex, education, and *APOEε4* allele count, and full model with principal components (Full Model + PC1-3). **(A)** Models using the entire NACC LOAD sample. Full model had greater results (AUC = 0.583) than PRS (*P*_Threshold_ = 0.005) alone (AUC = 0.527). **(B)** Similar analysis was done in an *APOEε3* homozygote sample to measure PRS effectiveness in a large dataset independent of *APOEε4*. Full model results were comparable to the full LOAD sample (AUC = 0.587), wheras PRS alone performed worse (AUC = 0.507).

To validate results from ROSMAP, the generated PRS (*P*_Threshold_ = 0.005) was tested in NACC dataset. The model that included only the PRS (*P*_Threshold_ = 0.005) showed an AUC of 0.527 and the full model showed an AUC of 0.581 (Figure 4A, Table 2 and Supplementary Table 15). In addition, the results of the full model demonstrated significant contributions from the PRS (beta = 0.010, *P* = 0.0194) as well as baseline age (beta = –0.016, *P* = 2e^–4^), and sex (beta = –0.231, *P* = 4.3e^–9^) (Supplementary Table 15). However, the DeLong test comparing the full model with full model excluding PRS (Supplementary Table 14) demonstrated no significant gain of model performance (*P* = 0.285; Supplementary Table 18). The full model was improved when adjusting for population structure by applying the PCA (AUC = 0.591; Table 2 and Supplementary Table 20). However, the PRS (*P*_Threshold_ = 0.005) had no significant contribution to the full model (beta = 0.068, *P* = 0.088). Additionally, the DeLong test demonstrated no significant model improving effects of the PRS comparing to the model without the PRS (*P* = 0.637; Supplementary Tables 19, 23).

We repeated the analysis using a subgroup of *APOEε3* homozygotes for the NACC cohort. The PRS (*P*_Threshold_ = 0.005) alone resulted in an AUC of 0.507, and application of the full model reached an AUC of 0.587 (Figure 4B, Table 2, and Supplementary Table 17). In this full model analysis, baseline age (beta = –0.018, *P* = 0.0049) and sex (beta = –0.251, *P* = 9e-5) showed significant effects (Figure 4B), but PRS (*P*_Threshold_ = 0.005) did not reach statistical significance (beta = –0.038, *P* = 0.551; Supplementary Table 17). Furthermore, the DeLong test demonstrated that the PRS (*P*_Threshold_ = 0.005) did not improve the model performance when comparing to the model excluding PRS (*P* = 0.362; Supplementary Tables 16, 18). When adding PC1-3 to account for population structure, prediction accuracy of the full model improved (AUC = 0.612; Table 2 and Supplementary Table 22), but the PRS (*P*_Threshold_ = 0.005) did not show a significant contribution (beta = -0.076, *P* = 0.253). Moreover, the PRS did not improve model performance when compared to a model without the PRS (*P* = 0.351; Supplementary Tables 21, 23).

### Predicting Time to Depression Onset

Identifying individuals at risk of developing depression earlier in the course of LOAD may serve to indicate severity of the disease, informing future treatment plans. Thus, time-to-event analysis was conducted to assess the PRS ability to predict those at risk for developing depression early in their LOAD trajectory by examining the time interval between age at entry of respective study and age at depression onset (or age at last visit if no depression occurred). We tested PRS calculated by the two formulas (see “Materials and Methods” section): (1) PRS and, (2) risk-increasing PRS. PRS uses the standard calculation approach, while risk-increasing PRS utilizes alleles with positive betas, or risk alleles, providing an interpretable score in terms of the number of risk alleles.

#### Rush Memory and Aging Project

Models using both formulas, PRS (*P*_Threshold_ = 0.005) and risk-increasing PRS (*P*_Threshold_ = 0.005), reached statistical significance (Table 3). In each of the full statistical models that included the covariates sex, baseline age, education and *APOEε4* allele count, the term for the PRS (beta = 0.146, *P* = 6e-4) and the risk-increasing PRS (beta = 0.006, *P* = 6e-4) had statistically significant effects in their respective models (Supplementary Tables 24, 25). Both PRS (*P*_Threshold_ = 0.005) and risk-increasing PRS (*P*_Threshold_ = 0.005) maintained significant contributions when adding PC1-3 to adjust for population structure (PRS: beta = 0.149, *P* = 0.001; risk-increasing PRS: beta = 0.006, *P* = 0.001; Supplementary Tables 28, 30). As age at baseline had a strong contribution to the full model (beta = –0.115, *P* = 1.7e-50), a model without age at baseline was subsequently calculated. The full models remained significant, with both PRS and risk-increasing PRS having significant contributions (PRS: beta = 0.141, *P* = 0.001; risk-increasing PRS: beta = 0.006, *P* = 0.001; Supplementary Tables 29, 31).

**TABLE 3:** Assessing both PRS and risk-increasing PRS ability to predict time interval of depression in LOAD.

	ROSMAP		NACC	
Sample	Full LOAD Sample	*APOEε3* Homozygote Sample	Full LOAD sample	*APOEε3* Homozygote Sample
Full model (*p*-value)	<0.001*	<0.001*	<0.001*	<0.001*
PRS alone (*p*-value)	0.005*	0.075	0.004*^a^*	0.025*^b^*
PRS: risk-ratio [95%CI]	1.126 [1.036, 1.222]	1.102 [0.990, 1.225]	0.991 [0.957, 1.027]	0.987 [0.930, 1.046]
Risk-increasing PRS: risk-ratio [95%CI]	1.005 [1.001, 1.008]	1.004 [1.000, 1.008]	0.999 [0.999, 0.9998]	0.999 [0.999, 0.9999]

*Full Model contained covariates of PRS, APOEε4 allele count, sex, baseline age, and education. Time-to-event analysis was performed using left-truncated (age at entry) and right censored (age at depression onset or age at last visit) data.*

*^a^p-value noted with risk-PRS. With standard PRS, p-value = 0.631.*

*^b^p-value noted with risk-PRS. With standard PRS, p-value = 0.658.*

*Statistical signifiance met.

*LOAD, Late-onset Alzheimer’s Disease; ROSMAP, Relgious Orders Study and Rush Memory and Aging Project; NACC, National Alzheimer’s Coordinating Center; APOE, Apolipoprotein E; SNP, Single Nucleotide Polymorphism; PRS, Polygenic Risk Score.*

Upon stratification for *APOEε3* homozygotes, the models using PRS and risk-increasing PRS (*P*_Threshold_ = 0.005), each alone, did not produce significant results (Table 3). Of note, the risk-increasing PRS performed comparably to its use in the full LOAD sample when comparing risk ratios. Nonetheless, the full models for both PRS formulas showed significant results (Table 3), with baseline age having a significant effect (Supplementary Tables 26, 27). Furthermore, both PRS (beta = 0.139, *P* = 0.011) and risk-increasing PRS (beta = 0.006, *P* = 0.010) had significant contributions to their respective full models (Supplementary Tables 26, 27, respectively). Both full models remained significant when adding PC1-3, with both PRS (beta = 0.137, *P* = 0.013) and risk-increasing PRS (beta = 0.005, *P* = 0.013) having significant contributions (Supplementary Tables 32, 34). However, after excluding age at baseline, both full models failed to meet significance (*P* = 0.058 for both). Nonetheless, both PRS (beta = 0.115, *P* = 0.035; Supplementary Table 33) and risk-increasing PRS (beta = 0.005, *P* = 0.035; Supplementary Table 35) had significant contributions to their respective models.

**FIGURE 5:**
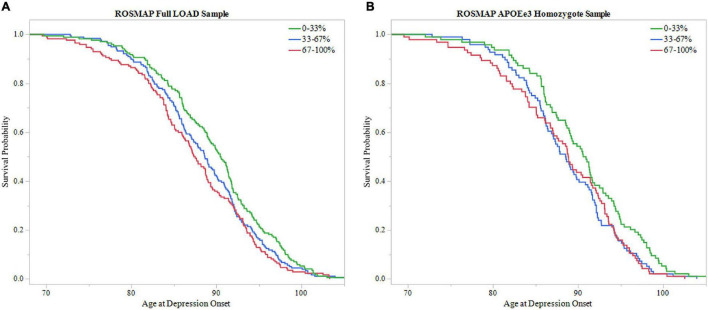
Survival analysis of PRS in ROSMAP. The sample was divided into three groups based on 0–33, 33–67, and 67–100% PRS distribution. The 0–33% group had low PRS scores indicating lower predicted risk of developing depression, the 67–100% group had higher PRS scores with higher predicted risk, and the 33–67% group falling in the middle. Survival curves were used to assess effect of higher PRS scores on age of depression onset. **(A)** Survival curves in the full LOAD sample demonstrated that individuals within the high PRS score group had earlier onsets of depression, meeting significance using the log-rank test (*P* = 0.019) **(B)** In the *APOEε3* homosygote sample, individuals within higher PRS score groups had eariler depression onset, meeting signficance with the log-rank test (*P* = 0.036).

Additionally, survival probability curves of both full LOAD and *APOEε3* homozygote samples were created (Figure 5). Each sample was divided into three groups based on PRS score distribution, 0–33, 33–67, and 67–100%. Both ROSMAP samples demonstrated that individuals with higher PRS scores had earlier age of depression onset, supported by the log-rank test (Full LOAD: *P* = 0.019, *APOEε3* homozygote: *P* = 0.036).

#### National Alzheimer’s Coordinating Center

We utilized PRS (*P*_Threshold_ = 0.005) and the risk-increasing PRS (*P*_Threshold_ = 0.005) in the time-to-event analyses. The model employing the PRS, alone, did not produce significant results. However, the risk-increasing PRS met statistical significance, but lacks practical utility with the 95% confidence interval of the risk ratio narrowly excluding 1 (*P* = 0.004; Table 3). The full models, using each of the two PRS formulas with other covariates, showed significant results (Table 3 and Supplementary Tables 36, 37). The full model using the PRS had significant contributions from baseline age, sex, education, and PRS (beta = 0.050, *P* = 0.009), while the full model using risk-increasing PRS had significant contributions from baseline age, education and risk-increasing PRS (beta = 0.0006, *P* = 0.003) (Supplementary Tables 36, 37, respectively). Both full models remained significant after adjusting for population structure by PCA, with PRS (beta = 0.050, *P* = 0.010) and risk-increasing PRS (beta = 0.0007, *P* = 0.002) continuing to have significant contributions to their respective models (Supplementary Tables 40, 42). Both full models sustained significance after removing age at baseline. The PRS did not have a significant effect on its full model (Supplementary Tables 36, 37), but the risk-increasing PRS had a significant effect (beta = –0.0006, *P* = 0.009; Supplementary Table 43), although in the negative direction.

Repeating the analyses for the *APOEε3* homozygote subgroup did not show significant results for PRS alone model. The risk-increasing PRS alone model saw significance, but the 95% confidence interval of the risk ratio narrowly excluded 1 (*P* = 0.025; Table 3). The full models for both PRS and risk-increasing PRS resulted in significant results (Supplementary Tables 38, 39) with major contributions from the covariates baseline age, sex, and education. However, neither the PRS nor the risk-increasing PRS had significant effects in their respective full models. Accounting for population structure, both full models reached significance, but neither PRS nor the risk-increasing PRS had significant contributions to their respective models (Supplementary Tables 44, 46). After excluding age at baseline, both full models maintained significance (Full model with: PRS *P* = 0.004, risk-increasing PRS *P* = 0.0008), with no significant effects from either the PRS or the risk-increasing PRS (Supplementary Tables 45, 47).

**FIGURE 6:**
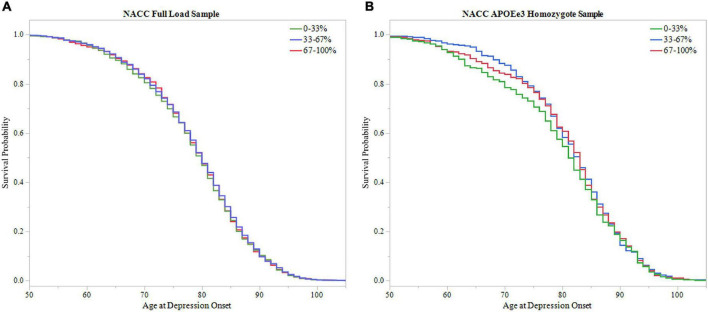
Survival analysis of PRS in NACC. The sample was divided into three groups based on 0–33, 33–67, and 67–100% PRS distribution. The 0–33% group had low PRS scores indicating lower predicted risk of developing depression, the 67–100% group had higher PRS scores with higher predicted risk, and the 33–67% group falling in the middle. Survival curves were used to assess effect of higher PRS scores on age of depression onset. **(A)** Survival curves in the full LOAD sample illustrated no effect of PRS score distribution group on age of depression onset, failing to meet signficance using the log-rank test (*P* = 0.704) **(B)** In the *APOEε3* homosygote sample, there was no signficanct effect of PRS score distribution group on age of depression onset using the log-rank test (*P* = 0.433).

To further assess PRS ability in predicting age of depression onset, survival probability curves were calculated for both NACC full LOAD and *APOEε3* homozygote samples (Figure 6). Each sample was broken into three groups based on PRS score distribution, 0–33, 33–67, and 67–100%. Both samples failed to meet significance using the log-rank test (Full LOAD: *P* = 0.704, *APOEε3* homozygote: *P* = 0.433), supporting time-to-event analysis finding non-significant results from the PRS alone models.

## Discussion

LOAD is a heterogenous disease with various genetic etiologies (Nacmias et al., 2018; Lo et al., 2019) and diverse phenotypes including: heterogeneity of biomarkers (Dujardin et al., 2020), coexisting pathologies (Lam et al., 2013), and clinical symptoms (Komarova and Thalhauser, 2011; Lam et al., 2013; Devi and Scheltens, 2018; Ferreira et al., 2018). Clinical heterogeneity is manifested also by comorbid neuropsychiatric symptoms (NPS), amongst which depression is very common. However, why some LOAD patients develop depression while others do not remain elusive. Previously, we found genetic pleiotropy between MDD and LOAD (Lutz et al., 2020), suggesting that genetics may contribute to the risk of depression symptom in LOAD. In this study to test this hypothesis, we performed the first genetic comparison analysis between LOAD patients with and without depression to explore the genetic heterogeneity of the risk and onset time of depression in individuals with LOAD. We derived a PRS that showed small to moderate effects in predicting depression onset in LOAD patients. That is to say, the PRS developed in our study resulted in an odds ratio (OR) of 1.1–1.4, which is small to moderate compared to the large effect size of OR = 3.09 for *APOEε*4 heterozygosity for LOAD risk (Neu et al., 2017). The PRS predictive ability was improved with the inclusion of the covariates age, sex, education, *APOEε4* allele count, with the addition of childhood financial need further enhancing the predictive performance of the model.

PRS are a well-established approach for the study of the genetics of complex diseases including LOAD and the utility of PRS to predict LOAD risk has been investigated by different groups (Escott-Price et al., 2015, 2017, 2019; Darst et al., 2017; Tasaki et al., 2018, 2019; Leonenko et al., 2019; Altmann et al., 2020; Tesi et al., 2020; Daunt et al., 2021; Zettergren et al., 2021). However, to our knowledge, this is the first study that progresses the use of PRS to predict clinical endophenotypes in LOAD, in particular depression. Our study is innovative in several ways: (1) The study was uniquely designed such that all subjects are LOAD patients where case-control status was defined by the manifestation of depression symptom. (2) Most prior LOAD PRS studies focused on LOAD prediction employing LOAD GWAS summary statistics. Here we tested the utility of PRS based on GWAS data from a particular disorder (MDD) to predict risk for a shared phenotype (depression) in individuals with another disorder (LOAD). (3) While previous work identified unique trajectories of depression and apathy in LOAD subjects and biomarkers associated with LOAD-specific depression progression (Banning et al., 2021), the current work focused on a genetic based prediction model of depression in LOAD. Collectively, our approach generated PRS to identify LOAD subjects with greater genetic risk of developing depression and those at risk to develop depression earlier in the time course of LOAD.

PRSs were calculated in ROSMAP and then replicated in NACC cohorts. The results obtained for the two cohorts were generally consistent showing a moderate predictivity of the PRS. However, there are some differences. In the NACC cohort, the PRS alone was more effective in classifying depression cases as evidenced by the logistic regression analysis, and it made more significant contributions to the full prediction model than in ROSMAP. However, the overall model performance was greater in ROSMAP. A possible explanation might be that ROSMAP is more homogenous than NACC, as ROSMAP contains a reduced range of baseline ages and is disproportionately female (Bennett et al., 2012a,b, 2018) resulting in greater homogeneity compared to NACC. Furthermore, the study selection criteria of ROSMAP may further contribute to the homogeneity in ROSMAP, with ROS enrolling priests and MAP selecting within the northeastern Illinois region. Therefore, the covariates, such as baseline age and education, would be expected to have greater effects in ROSMAP. Thus, statistical models would be expected to show predictive ability that would appear stronger in ROSMAP. NACC’s diversity and sample size led to greater classification ability, and greater contribution in the full model. In both datasets, the PRS did not add significant improvement to model performance, with the greatest statistical increase in performance attributable to PRS observed in the full LOAD NACC sample. As the PRS had promising results in two different criteria, prediction and classification, the PRS demonstrated generalizability, although with small effects. Similar results were observed for the time-to-event analysis, which tested PRS ability to distinguish individuals more at risk of developing depression earlier in their LOAD trajectory. A risk-increasing PRS was calculated to provide an interpretable score, where a unit increase in risk-increasing PRS corresponds to an additional risk allele. In ROSMAP, both PRS versions performed effectively, both as standalone models and within the full models. In NACC, both PRS types had significant contributions to their respective full statistical models, but not as PRS term only models.

*APOEε4* genotype, is the strongest genetic risk factor for LOAD. *APOE* genotype may influence not only LOAD risk and age of onset but also the disease severity, progression, and the presence of certain comorbid endophenotypes, including NPS. For example, several studies have found associations between increased risk for depression and the *APOEε4* allele (Geda et al., 2006; Feng et al., 2015; Wang et al., 2019), while other studies have not replicated these findings (Slifer et al., 2009). To consider the potential confounding effect of *APOEε4* genotype on the occurrence of depression in LOAD, we repeated the analyses with sub samples of *APOEε3* homozygotes only. In terms of predicting depression onset, the PRS saw a slight decline in predictive performance relative to the analysis that included all *APOE* genotypes for the ROSMAP sample and equivalent performance in the NACC sample. The full model including childhood financial need in ROSMAP lead to the most predictive model, with a moderate effect of the childhood financial need variable. This result highlights that childhood struggles may translate into risk of depression later in life, consistent with previous report that childhood adversity have lower psychosocial capabilities late in life (Wilson et al., 2006). Additionally, the moderate effect of childhood financial need points to environmental factors contributing to depression risk. Unfortunately, this variable was not available in the NACC cohort for further assessments. The time-to-event analysis did not find significant results from standalone PRS models. However, in ROSMAP, both PRS types played significant roles in the full models. The *APOEε3* homozygote study demonstrated that *APOEε4* does not have a significant role in the risk of depression onset in individuals with LOAD, with small declines in PRS performance and stable or improved full model results.

## Limitations

Our study has some limitations. First, the scales used to assess depression were different among ROSMAP and NACC, which introduced challenges to compare between the cohorts. Further, the treatment of depression in ROSMAP participants increased the number of participants defined as co-occurring depression cases. However, treating depression to determine case-control status resulted in similar proportions of depression cases in the two datasets. Second, the binary treatment of depression introduced another limitation. The current method treats depression as either being present or not present throughout the individual’s course in either ROSMAP or NACC. This fails to account for the possibility that depression may occur numerous times within the course of the study and might have varying degrees of severity. Further work can expand upon the number of instances of depression to enrich genetic models. Additionally, sub-threshold depression symptoms may confound the results, as some control subjects may manifest relatively mild depressive symptoms and thus were undiagnosed. The method ROSMAP coded depression had more endpoints for depression, allowing us to overcome, at least in part, this limitation. Third, the current study focusses on depression comorbid with LOAD. Thus, this study is unable to explore the role of depression prior to LOAD diagnosis. Future study would include depression history preceding to LOAD diagnosis and examine its possible confounding effect. Fourth, not all SNP genotypes were available in both datasets, which introduced a challenge to compare the results between ROSMAP and NACC. Fifth, the ROSMAP participants are included within the NACC cohort. While it was rigorous to exclude the ROSMAP samples from the NACC validation cohort, due to privacy concerns the ROSMAP subjects were not identifiable and therefore it was not possible to exclude them from the larger NACC cohort. However, the ROSMAP sample represents only a small group within the larger sample size of the NACC cohort. Sixth, the ROSMAP and NACC cohorts differ greatly in terms of sample size and demographic characteristic. Therefore, comparing PRS effects between these datasets has inherent limitations, nonetheless, on the other hand, it demonstrated the generalizability of the PRS performance within a European ancestry sample. Seventh, PRS was only tested in primarily European ancestry individuals and thus results may not hold true for other ancestries. Eighth, while we viewed this work as a continuation of our previous study (Lutz et al., 2020), and thus used the same MDD GWAS summary statistics (Wray et al., 2018) to construct the PRS, we acknowledge that two new MDD GWAS were published. The first is a European ancestry MDD GWAS (Howard et al., 2019) and the second is an East Asian ancestry MDD GWAS (Giannakopoulou et al., 2021). These studies will be used in future work to evaluate the risk of depression in LOAD and to replicate our findings. Ninth, the PRS and other covariates had small effect sizes, especially for models tested in NACC. The smaller estimated effects resulting from models using NACC data, could be due to larger, genetically heterogenous data. Despite small estimates in NACC, the PRS made significant contributions. Nevertheless, this study advances the current work on PRS and further explores the performance of MDD genetic factors in predicting risk of depression development in LOAD subjects.

## Conclusion

The results of this study indicate that the PRS for depression is an effective genetic tool to predict risk or onset time interval for depression in individuals with LOAD. This would facilitate greater prognostic capabilities to assess LOAD patients with potential disease trajectories predisposing depression. Furthermore, current prescribed antidepressants in patients with dementia show little or no effect on depressive symptoms, cognitive functioning, and activities of daily living in LOAD patients. Additionally, some antidepressants might even cause adverse events (Lanctôt et al., 2017; Dafsari and Jessen, 2020). Thus, the treatment of depression in LOAD needs a critical approach. Due to this lack of effective anti-depressants for LOAD patients, the importance of this study lies in the ability of the present depression PRS to enrich future clinical trials tasked with identifying a potential new antidepressant treatment for LOAD patients. Importantly, additional studies are needed to confirm and replicate our findings prior to progressing the use of the depression risk PRS towards clinical settings.

## Data Availability Statement

Publicly available datasets were analyzed in this study. The MDD GWAS summary statistics are available at: https://www.med.unc.edu/pgc/results-anddownLOADs/mdd/. The ROSMAP data can be requested at: https://www.radc.rush.edu/. The NACC data can be requested at: https://naccdata.org/requesting-data/submit-data-request. The PRS calculation code can be found at: https://github.com/surajupad/Depression-PRS.git.

## Author Contributions

OC-F and ML contributed to conception and design of the study. SU performed the polygenic risk score creation and statistical analysis, with assistance from ML, HL, and OC-F. HL and SL provided NACC data. SU, ML, and OC-F interpreted the result. SU wrote the first draft of the manuscript. SU, ML, SL, HL, and OC-F contributed to manuscript revision, read, and approved the submitted version. OC-F obtained funding. All authors contributed to the article and approved the submitted version.

## Conflict of Interest

The authors declare that the research was conducted in the absence of any commercial or financial relationships that could be construed as a potential conflict of interest.

## Publisher’s Note

All claims expressed in this article are solely those of the authors and do not necessarily represent those of their affiliated organizations, or those of the publisher, the editors and the reviewers. Any product that may be evaluated in this article, or claim that may be made by its manufacturer, is not guaranteed or endorsed by the publisher.

## Footnotes

1
https://imputationserver.sph.umich.edu

## Abbreviations

LOAD: Late-onset Alzheimer’s Disease
PRS: Polygenic Risk Score
CSF: Cerebrospinal Fluid
GWAS: Genome-Wide Association Studies
MDD: Major Depressive Disorder
*APOE*: Apolipoprotein E
NPS: Neuropschiatric Symptoms
MCI: Mild Cognitive Impariment
ROSMAP: Relgious Orders Study and Rush Memory and Aging Project
NACC: National Alzheimer’s Coordinating Center
SNP: Single Nucleotide Polymorphism
AUC: Area Under the Cruve
DSM-III: Diagnostic and Statistcal Manual of Mental Disorders 3rd edition
GDS: Geriatric Dementia Scale
ADRC: Alzheimer’s Disease Research Center
MAF: Minor Allele Frequency
SD: Standard Deviation
PC: Principal Component.
