# Supplementary material for: Polygenic Risk Score Effectively Predicts Depression Onset in Alzheimer’s Disease Based on Major Depressive Disorder Risk Variants
Source: Front Neurosci. 2022 Mar 8;16:827447. doi: 10.3389/fnins.2022.827447 (PMC8957806; doi:10.3389/fnins.2022.827447)
Supplement: Supplementary file 1 [file Data_Sheet_1.pdf]

Polygenic risk score effectively predicts risk of depression onset in Alzheimer's disease

Supplementary Material

Table S1: SNP Counts

P-value threshold[1]	ROSMAP	NACC
0.5	191077	163667
0.4	160692	140498
0.3	128917	114531
0.2	94611	85311
0.1	55554	50654
0.05	32671	29886
0.01	9785	8916
0.005	5915	5271
0.001	1841	1587
0.0001	420	374
10^{-5}	96	96
10^{-6}	32	39
10^{-7}	16	21
10^{-8}	7	12

[1]SNP selection threshold

Table S2: Case-Control parameter estimates in ROSMAP

PRS	Estimate[1]	Std Error[1]	P[1]
PRS ($P_{Threshold}$=0.5)	0.062	0.090	0.493
PRS ($P_{Threshold}$=0.4)	0.076	0.090	0.397
PRS ($P_{Threshold}$=0.3)	0.085	0.091	0.348
PRS ($P_{Threshold}$=0.2)	0.143	0.093	0.124
PRS ($P_{Threshold}$=0.1)	0.116	0.092	0.207
PRS ($P_{Threshold}$=0.05)	0.136	0.090	0.130
PRS ($P_{Threshold}$=0.01)	0.101	0.090	0.261
PRS ($P_{Threshold}$=0.005)*	0.153	0.090	0.089
PRS ($P_{Threshold}$=0.001)	0.110	0.090	0.222
PRS ($P_{Threshold}$=0.0001)	-0.065	0.094	0.489
PRS ($P_{Threshold}$=1e-05)	-0.042	0.094	0.656
PRS ($P_{Threshold}$=1e-06)	-0.014	0.094	0.881
PRS ($P_{Threshold}$=1e-07)	0.019	0.093	0.842
PRS ($P_{Threshold}$=1e-08)	-0.007	0.090	0.936

[1]Logistic regression analysis using the PRS made at different SNP thresholds in the ROSMAP dataset.

* PRS ($P_{Threshold}$=0.005) was deemed optimal and used for subsequent analysis in ROSMAP.

Table S3: Case-Control parameter estimates in NACC

PRS	Estimate[1]	Std Error[1]	P[1]
PRS ($P_{Threshold}$=0.5)	0.114	0.039	**0.0033**
PRS ($P_{Threshold}$=0.4)	0.108	0.039	**0.0052**
PRS ($P_{Threshold}$=0.3)	0.116	0.039	**0.0028**
PRS ($P_{Threshold}$=0.2)	0.113	0.039	**0.0034**
PRS ($P_{Threshold}$=0.1)	0.078	0.038	**0.0407**
PRS ($P_{Threshold}$=0.05)	0.088	0.039	**0.0227**
PRS ($P_{Threshold}$=0.01)	0.093	0.038	**0.0159**

PRS ($P_{Threshold}$=0.005)*	0.092	0.038	**0.0149**
PRS ($P_{Threshold}$=0.001)	0.112	0.038	**0.0031**
PRS ($P_{Threshold}$=0.0001)	0.048	0.038	0.2161
PRS ($P_{Threshold}$=1e-05)	0.040	0.038	0.2910
PRS ($P_{Threshold}$=1e-06)	0.056	0.039	0.143
PRS ($P_{Threshold}$=1e-07)	0.054	0.039	0.162
PRS ($P_{Threshold}$=1e-08)	0.049	0.039	0.204

[1]Logistic regression analysis using the PRS made at different SNP thresholds in the NACC dataset.

*PRS ($P_{Threshold}$=0.005) was employed in further analysis in NACC, to validate ROSMAP results.

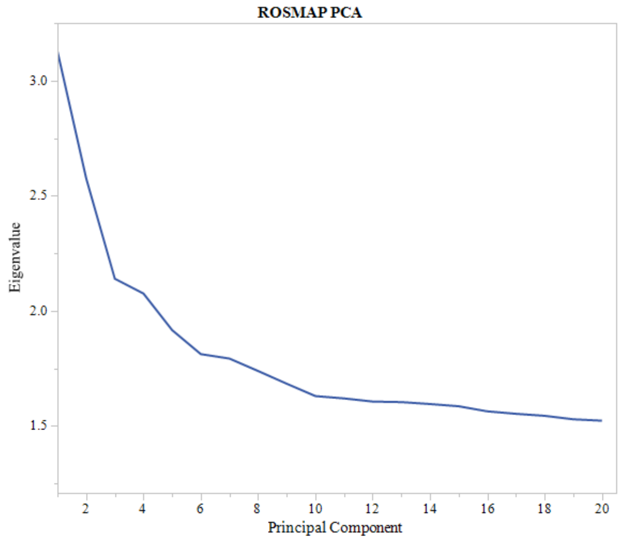

Figure S1: Scree plot of ROSMAP Principal Component Analysis. Eigenvalues overall were low in the ROSMAP sample. This indicates overall homogeneity of the sample. The first three principal components (PC) were selected as covariates for subsequent analysis.

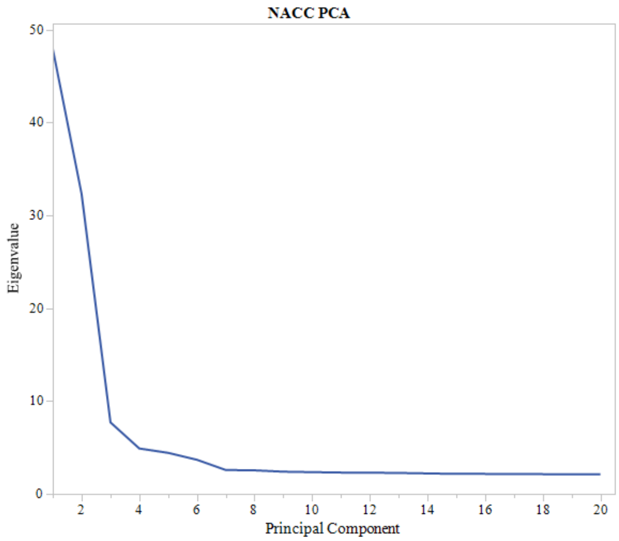

Figure S2: Scree plot of NACC Principal Component Analysis. NACC saw greater eigenvalues, which would represent greater heterogeneity of population structure. The first three principal components (PC) were selected for subsequent analysis.

Table S4: Full Model, excluding PRS, parameter estimates using ROC analysis in ROSMAP

Term	Estimate [95% CI][1]	Std Error[1]	P[1]
Intercept	6.245 [0.969, 11.822]	2.754	**0.023**
Sex (Female)	0.057 [-0.332 0.450]	0.198	0.775
Age at Baseline	-0.062 [-0.126, -0.001]	0.032	**0.050**
Education	-0.125 [-0.248, -0.006]	0.062	**0.043**
APOEe4[0][2]	0.518 [-0.180, 1.279]	0.358	0.148
APOEe4[1][2]	-0.409 [-1.166, 0.388]	0.384	0.287
Financial Need	0.100 [-0.164, 0.364]	0.133	0.452

[1]Multivariate logistic regression using baseline age, sex, years of education, childhood financial need, and *APOEε4* allele count.

[2]Subjects with two *APOEε4* allele copies serve as the reference group.

Table S5: Full Model parameter estimates using ROC analysis in ROSMAP

Term	Estimate [95% CI][1]	Std Error[1]	P[1]
Intercept	5.872 [0.516, 11.522]	2.792	**0.035**

PRS ($P_{\text{Threshold}}$=0.005)*	0.249 [-0.067, 0.574]	0.163	0.126
Age at Baseline	-0.056 [-0.121, 0.006]	0.032	0.082
Sex (female)	0.037 [-0.355, 0.434]	0.200	0.852
Education	-0.134 [-0.260, -0.013]	0.063	**0.033**
Financial Need	0.143 [-0.132, 0.418]	0.139	0.306
APOEe4[0][2]	0.498 [-0.208, 1.266]	0.362	0.169
APOEe4[1][2]	-0.447 [-1.215, 0.357]	0.389	0.251

[1]Multivariate logistic regression using PRS ($P_{\text{Threshold}}$=0.005), baseline age, sex, years of education, childhood financial need, and *APOEε4* allele count.

[2]Subjects with two *APOEε4* allele copies serve as the reference group.

* $P_{\text{Threshold}}$=0.005 refers to the p-value threshold used to select SNPs for PRS generation

Table S6: Full model, excluding PRS, parameter estimates using ROC analysis in a ROSMAP APOEε3 homozygote sample

Term	Estimate [95% CI][1]	Std Error[1]	P[1]
Intercept	11.184 [3.733, 19.591]	4.004	**0.005**
Sex (Female)	0.008 [-0.534, 0.557]	0.276	0.978
Age at Baseline	-0.122 [-0.218, -0.037]	0.046	**0.008**
Education	-0.103 [-0.276, 0.065]	0.086	0.234
Financial Need	0.520 [0.115, 0.989]	0.219	**0.017**

[1]Multivariate logistic regression using baseline age, sex, years of education, and childhood financial need.

Table S7: Full Model parameter estimates using ROC analysis in a ROSMAP APOEε3 homozygote sample

Term	Estimate [95% CI][1]	Std Error[1]	P[1]
Intercept	10.712 [3.140, 19.244]	4.064	**0.0084**
PRS ($P_{\text{Threshold}}$=0.005)*	0.349 [-0.104, 0.828]	0.235	0.138

Education	-0.116 [-0.293, 0.053]	0.087	0.1855
Financial Need	0.595 [0.168, 1.083]	0.230	**0.0097**
Sex (Female)	-0.069 [-0.635, 0.496]	0.286	0.809
Age at Baseline	-0.115 [-0.212, -0.027]	0.047	**0.0139**

[1]Multivariate logistic regression using PRS ($P_{\text{Threshold}}$=0.005), baseline age, sex, years of education, and childhood financial need.

[*] $P_{\text{Threshold}}$=0.005 refers to the p-value threshold used to select SNPs for PRS generation

Table S8: Pairwise comparison of ROC curves in full LOAD ROSMAP sample

**Comparisons**	**AUCs of both Models**	**Difference in AUC**	**Std Error[1]**	**P[1]**
Full LOAD Sample				
Model[2] without PRS[3] vs Model with PRS	0.603 vs 0.606	0.003	0.010	0.774
Model[2] including childhood financial need without PRS vs Model including childhood financial need with PRS	0.663 vs 0.680	0.017	0.020	0.377
APOEε3 homozygote sample				
Model[4] without PRS[3] vs Model with PRS	0.615 vs 0.624	0.010	0.011	0.377
Model[4] including childhood financial need without PRS vs Model including childhood financial need with PRS	0.692 vs 0.721	0.029	0.024	0.237

[1]DeLong test[1]

[2]Model consisted of baseline age, sex, years of education, and *APOEε4* allele count

[3]PRS ($P_{\text{Threshold}}$=0.005)

[4]Model consisted of baseline age, sex, and years of education

Table S9: Full Model parameter estimates, without PRS, ROC analysis in ROSMAP adjusting with PC1-3

**Term**	**Estimate [95% CI][1]**	**Std Error[1]**	**P[1]**

Intercept	5.312 [-0.152, 11.051]	2.842	0.062
Age at Baseline	-0.053 [-0.118, 0.010]	0.033	0.105
Sex (Female)	0.033 [-0.361, 0.431]	0.201	0.871
Education	-0.110 [-0.235, 0.011]	0.062	0.078
APOE4[0][2]	0.504 [-0.208, 1.277]	0.366	0.168
APOE4[1][2]	-0.446 [-1.221, 0.368]	0.394	0.258
Financial need	0.091 [-0.183, 0.362]	0.138	0.509
PC1	-24.216 [-56.791, 7.266]	16.241	0.136
PC2	-73.118 [-202.511, 53.536]	64.945	0.260
PC3	1.718 [-11.982, 15.346]	6.892	0.803

[1]Multivariate logistic regression using baseline age, sex, years of education, childhood financial need, *APOEε4* allele count, PC1, PC2, and PC3.

[2]Subjects with two *APOEε4* allele copies serve as the reference group.

Table S10: Full Model parameter estimates using ROC analysis in ROSMAP adjusting with PC1-3

Term	Estimate [95% CI][1]	Std Error[1]	P[1]
Intercept	4.982 [-0.587, 10.820]	2.894	0.085
PRS ($P_{Threshold}$=0.005)*	0.250 [-0.071, 0.579]	0.165	0.129
Age at Baseline	-0.047 [-0.114, 0.017]	0.033	0.155
Sex (Female)	0.009 [-0.389, 0.411]	0.203	0.964
Education	-0.120 [-0.248, 0.003]	0.064	0.060
APOE4[0]	0.480 [-0.250, 1.270]	0.375	0.201
APOE4[1]	-0.486 [-1.282, 0.345]	0.404	0.228
Financial Need	0.141 [-0.145, 0.426]	0.145	0.331

PC1	-22.243 [-55.142, 9.766]	16.457	0.177
PC2	-60.428 [-192.220, 69.151]	66.302	0.362
PC3	2.335 [-11.459, 16.167]	6.966	0.737

[1]Multivariate logistic regression using PRS ($P_{\text{Threshold}}$=0.005), baseline age, sex, years of education, childhood financial need, *APOEε4* allele count, PC1, PC2, and PC3.

[2]Subjects with two *APOEε4* allele copies serve as the reference group.

[*] $P_{\text{Threshold}}$=0.005 refers to the p-value threshold used to select SNPs for PRS generation

Table S11: Full Model adjusted with PC1-3 without PRS parameter estimates using ROC analysis in a ROSMAP APOEε3 homozygote sample

Term	Estimate [95% CI][1]	Std Error[1]	P[1]
Intercept	10.799 [3.035, 19.464]	4.147	**0.009**
Age at Baseline	-0.121 [-0.220, -0.032]	0.0476	**0.011**
Sex (Female)	0.001 [-0.542, 0.554]	0.277	0.997
Education	-0.092 [-0.268, 0.079]	0.088	0.295
Financial need	0.556 [0.134, 1.045]	0.228	**0.015**
PC1	-4.047 [-51.561, 42.387]	23.670	0.864
PC2	10.415 [-178.182, 196.926]	94.575	0.912
PC3	8.456 [-9.561, 26.849]	9.131	0.354

[1]Multivariate logistic regression model using baseline age, sex, years of education, childhood financial need, PC1, PC2, and PC3.

Table S12: Full Model adjusted with PC1-3 parameter estimates using ROC analysis in a ROSMAP APOEε3 homozygote sample

Term	Estimate [95% CI][1]	Std Error[1]	P[1]
Intercept	10.254 [2.315, 19.088]	4.230	**0.015**

PRS ($P_{Threshold}$=0.005)*	0.385 [-0.076, 0.877]	0.241	0.110
Age at Baseline	-0.113 [-0.214, -0.021]	0.049	**0.021**
Sex (Female)	-0.085 [-0.657, 0.486]	0.289	0.769
Education	-0.104 [-0.285, 0.069]	0.089	0.244
Financial need	0.644 [0.199, 1.153]	0.240	**0.007**
PC1	-4.167 [-52.863, 43.465]	24.249	0.864
PC2	14.708 [-179.240, 207.106]	97.311	0.880
PC3	9.798 [-8.642, 29.026]	9.429	0.299

[1]Multivariate logistic regression model using PRS ($P_{Threshold}$=0.005), baseline age, sex, years of education, childhood financial need, PC1, PC2, and PC3.

* $P_{Threshold}$=0.005 refers to the p-value threshold used to select SNPs for PRS generation

Table S13: Pairwise comparison of ROC curves adjusted for PC1-3 in full LOAD ROSMAP sample

**Comparisons**	**AUCs of both Models**	**Difference in AUC**	**Std Error**[1]	**P**[1]
Full LOAD Sample				
Model[2] without PRS[3] vs Model with PRS	0.627 vs 0.629	0.002	0.009	0.854
Model[2] including childhood financial need without PRS vs Model including childhood financial need with PRS	0.692 vs 0.704	0.012	0.018	0.522
APOEε3 homozygote sample				
Model[4] without PRS[3] vs Model with PRS	0.637 vs 0.648	0.012	0.009	0.200
Model[4] including childhood financial need without PRS vs Model including childhood financial need with PRS	0.705 vs 0.728	0.023	0.025	0.361

[1]DeLong test[1]

[2]Model consisted of baseline age, sex, years of education, *APOEε4* allele count, PC1, PC2, and PC3

[3]PRS ($P_{Threshold}$=0.005)

[4]Model consisted of baseline age, sex, years of education, PC1, PC2, and PC3

Table S14: Full model, excluding PRS, parameter estimates using ROC analysis in NACC

Term	Estimate [95% CI][1]	Std Error[1]	P[1]
Intercept	0.655 [-0.021, 1.334]	0.346	0.058
Education	-0.002 [-0.013, 0.009]	0.006	0.752
Age at Baseline	-0.016 [-0.025, -0.008]	0.004	**2e-4**
Sex (male)	-0.232 [-0.309, -0.156]	0.039	**2.34e-9**
APOEe4[0][2]	0.1113 [-0.004, 0.227]	0.059	0.059
APOEe4[1][2]	0.0182 [-0.092, 0.129]	0.056	0.746

[1]Multivariate logistic regression using baseline age, sex, years of education, and *APOEε4* allele count.

[2]Subjects with two *APOEε4* allele copies serve as the reference group.

Table S15: Full Model parameter estimates using ROC analysis in NACC

Term	Estimate [95% CI][1]	Std Error[1]	P[1]
Intercept	0.650 [-0.028, 1.328]	0.346	0.0604
PRS ($P_{Threshold}$=0.005)*	0.010 [0.016, 0.165]	0.038	**0.0194**
Education	-0.001 [-0.013, 0.009]	0.006	0.79
Sex (male)	-0.231 [-0.307, -0.155]	0.039	**4.3e-9**
Age at Baseline	-0.016 [-0.025, -0.008]	0.004	**0.0002**
APOEe4[0][2]	0.109 [-0.006, 0.225]	0.059	0.0638
APOEe4[1][2]	0.012 [-0.098, 0.123]	0.056	0.829

[1]Multivariate logistic regression using PRS ($P_{Threshold}$=0.005), baseline age, sex, years of education, and *APOEε4* allele count.

[2]Subjects with two *APOEε4* allele copies serve as the reference group.

* $P_{Threshold}$=0.005 refers to the p-value threshold used to select SNPs for PRS generation

Table S16: Full model, excluding PRS, parameter estimates using ROC analysis in a NACC APOEε3 homozygote sample

Term	Estimate [95% CI][1]	Std Error[1]	P[1]
Intercept	1.023 [-0.003, 2.064]	0.526	**0.052**
Age at Baseline	-0.018 [-0.031, -0.006]	0.006	**0.005**
Education	-0.009 [-0.030, 0.009]	0.010	0.362
Sex (male)	-0.249 [-0.375, -0.124]	0.064	**1e-4**

[1]Multivariate logistic regression using baseline age, sex, and years of education.

Table S17: Full Model parameter estimates using ROC analysis in a NACC APOEε3 homozygote sample

Term	Estimate [95% CI][1]	Std Error[1]	P[1]
Intercept	1.023 [-0.003, 2.064]	0.526	0.0519
PRS ($P_{\text{Threshold}}$=0.005)*	-0.0382023 [-0.164, 0.087]	0.064	0.551
Education	-0.009 [-0.030, 0.009]	0.010	0.352
Sex (male)	-0.251 [-0.377, -0.126]	0.064	**9.1e-5**
Age at Baseline	-0.018 [-0.030, -0.005]	0.006	**0.0049**

[1]Multivariate logistic regression using PRS ($P_{\text{Threshold}}$=0.005), baseline age, sex, and years of education.

* $P_{\text{Threshold}}$=0.005 refers to the p-value threshold used to select SNPs for PRS generation

Table S18: Pairwise comparison of ROC curves in NACC full LOAD and APOEε3 homozygote samples

Comparisons	AUCs of both Models	Difference in AUC	Std Error[1]	P[1]
Full LOAD Sample				
Model[2] without PRS ($P_{\text{Threshold}}$=0.005) vs Model with PRS ($P_{\text{Threshold}}$=0.005)	0.577 vs 0.581	0.004	0.004	0.285
APOEε3 homozygote sample				

Model[3] without PRS ($P_{\text{Threshold}}$=0.005) vs Model with PRS ($P_{\text{Threshold}}$=0.005)	0.584 vs 0.587	0.002	0.002	0.362

[1]DeLong test

[2]Model consisted of baseline age, sex, years of education, and *APOEε4* allele count

[3]Model consisted of baseline age, sex, and years of education

Table S19: Full Model excluding PRS parameter estimates using ROC analysis in NACC adjusting with PC1-3

Term	Estimate [95% CI][1]	Std Error[1]	P[1]
Intercept	0.836 [0.152, 1.523]	0.350	**0.017**
Age at Baseline	-0.018 [-0.027, -0.010]	0.004	**3e-4**
Sex (male)	-0.237 [-0.313, -0.160]	0.039	**1.13e-9**
Education	-0.003 [-0.015, 0.008]	0.006	0.598
APOEe4[0][2]	0.102 [-0.015, 0.219]	0.060	0.087
APOEe4[1][2]	0.024 [-0.087, 0.135]	0.057	0.673
PC1	-3.972 [-25.333, 20.874]	11.1563	0.722
PC2	16.683 [6.793, 27.122]	5.109	**0.001**
PC3	3.445 [-8.290, 15.308]	6.002	0.566

[1]Multivariate logistic regression using baseline age, sex, years of education, *APOEε4* allele count, PC1, PC2, and PC3.

[2]Subjects with two *APOEε4* allele copies serve as the reference group.

Table S20: Full Model parameter estimates using ROC analysis in NACC adjusting with PC1-3

Term	Estimate [95% CI][1]	Std Error[1]	P[1]
Intercept	0.834 [0.150, 1.522]	0.350	**0.017**
PRS ($P_{\text{Threshold}}$=0.005)*	0.068 [-0.010, 0.147]	0.040	0.088

Age at Baseline	-0.018 [-0.027, -0.010]	0.004	**3e-4**
Sex (male)	-0.235 [-0.312, -0.159]	0.039	**1.48e-9**
Education	-0.002 [-0.015, 0.008]	0.006	0.626
APOEe4[0][2]	0.101 [-0.015, 0.218]	0.060	0.089
APOEe4[1][2]	0.020 [-0.091, 0.131]	0.057	0.730
PC1	0.106 [-21.738, 25.398]	11.429	0.993
PC2	16.663 [6.767, 27.122]	5.116	**0.001**
PC3	3.830 [-7.937, 15.720]	6.017	0.524

[1]Multivariate logistic regression using PRS ($P_{Threshold}$=0.005), baseline age, sex, years of education, *APOEε4* allele count, PC1, PC2, and PC3.

[2]Subjects with two *APOEε4* allele copies serve as the reference group.

[*] $P_{Threshold}$=0.005 refers to the p-value threshold used to select SNPs for PRS generation

Table S21: Full Model adjusted with PC1-3 excluding PRS parameter estimates using ROC analysis in a NACC APOEε3 homozygote sample

Term	Estimate [95% CI][1]	Std Error[1]	P[1]
Intercept	1.285 [0.241, 2.348]	0.536	**0.017**
Age at Baseline	-0.020 [-0.032, -0.007]	0.006	**0.002**
Education	-0.012 [-0.034, 0.006]	0.010	0.233
Sex (male)	-0.258 [-0.385, -0.131]	0.065	**6e-4**
PC1	-137.500 [-319.965, 37.464]	90.872	0.130
PC2	-23.096 [-80.975, 32.732]	28.918	0.425
PC3	36.130 [4.684, 68.742]	16.305	**0.027**

[1]Multivariate logistic regression using baseline age, sex, years of education, PC1, PC2, and PC3.

Table S22: Full Model adjusted with PC1-3 parameter estimates using ROC analysis in a NACC APOEε3 homozygote sample

Term	Estimate [95% CI][1]	Std Error[1]	P[1]
Intercept	1.288 [0.244, 2.353]	0.537	**0.016**
PRS ($P_{\text{Threshold}}$=0.005)*	-0.076 [-0.206, 0.054]	0.066	0.253
Age at Baseline	-0.020 [-0.032, -0.007]	0.006	**0.003**
Education	-0.012 [-0.035, 0.006]	0.010	0.216
Sex (male)	-0.262 [-0.389, -0.135]	0.065	**5e-4**
PC1	-140.380 [-323.394, 35.026]	91.125	0.123
PC2	-22.334 [-80.362, 33.640]	28.994	0.441
PC3	35.450 [3.929, 68.138]	16.343	**0.030**

[1]Multivariate logistic regression using PRS ($P_{\text{Threshold}}$=0.005), baseline age, sex, years of education, PC1, PC2, and PC3.

* $P_{\text{Threshold}}$=0.005 refers to the p-value threshold used to select SNPs for PRS generation

Table S23: Pairwise comparison of ROC curves adjusted for PC1-3 in NACC full LOAD and APOEε3 homozygote samples

Comparisons	AUCs of both Models	Difference in AUC	Std Error[1]	P[1]
Full LOAD Sample				
Model[2] without PRS ($P_{\text{Threshold}}$=0.005) vs Model with PRS ($P_{\text{Threshold}}$=0.005)	0.590 vs 0.591	0.001	0.002	0.637
APOEε3 homozygote sample				
Model[3] without PRS ($P_{\text{Threshold}}$=0.005) vs Model with PRS ($P_{\text{Threshold}}$=0.005)	0.609 vs 0.612	0.003	0.003	0.351

[1]DeLong test

[2]Model consisted of baseline age, sex, years of education, *APOEε4* allele count, PC1, PC2, and PC3

[3]Model consisted of baseline age, sex, years of education, PC1, PC2, and PC3

Table S24: Full model parameter estimates of the time-to-event analysis in ROSMAP

Term	Estimate [95% CI][1]	Std Error[1]	P[1]
Age at Baseline	-0.113 [-0.128, -0.098]	0.008	**3.25e-50**
Sex (female)	-0.113 [-0.207, -0.016]	0.049	**0.0224**
PRS ($P_{Threshold}$=0.005)*	0.146 [0.063, 0.229]	0.042	**0.0006**
Education	-0.043 [-0.069, -0.017]	0.013	**0.0011**
APOEe4[0][2]	-0.312 [-0.496, -0.105]	0.099	**0.0029**
APOEe4[1][2]	-0.038 [-0.231, 0.174]	0.103	**0.0029**

[1]Multivariate Cox Proportional Hazard model using PRS ($P_{Threshold}$=0.005), baseline age, sex, years of education, and *APOEε4* allele count.

[2]Subjects with two *APOEε4* allele copies serve as the reference group.

* $P_{Threshold}$=0.005 refers to the p-value threshold used to select SNPs for PRS generation

Table S25: Full model parameter estimates of the time-to-event analysis with the risk-increasing PRS in ROSMAP

Term	Estimate [95% CI][1]	Std Error[1]	P[1]
Age at Baseline	-0.113 [-0.128, -0.098]	0.008	**3.25e-50**
Sex (female)	-0.113 [-0.208, -0.017]	0.049	**0.0224**
Education	-0.043 [-0.069, -0.017]	0.013	**0.0011**
APOEe4[0][2]	-0.312 [-0.506, -0.117]	0.099	**0.0029**
APOEe4[1][2]	-0.038 [-0.240, 0.164]	0.103	**0.0029**
Risk-increasing PRS ($P_{Threshold}$=0.005)*	0.006 [0.003, 0.009]	0.002	**0.0006**

[1]Multivariate Cox Proportional Hazard model using risk-increasing PRS ($P_{Threshold}$=0.005), baseline age, sex, years of education, and *APOEε4* allele count.

[2]Subjects with two *APOEε4* allele copies serve as the reference group.

* $P_{Threshold}$=0.005 refers to the p-value threshold used to select SNPs for PRS generation

Table S26: Full model parameter estimates of the time-to-event analysis in a ROSMAP APOEε3 homozygote sample

Term	Estimate [95% CI][1]	Std Error[1]	P[1]
PRS ($P_{Threshold}$=0.005)*	0.139 [0.033, 0.245]	0.054	**0.0105**
Education	-0.025 [-0.060, 0.010]	0.018	0.1542
Age at Baseline	-0.097 [-0.117, -0.077]	0.010	**9.77e-22**
Sex (female)	-0.123 [-0.253, 0.012]	0.068	0.0735

[1]Multivariate Cox Proportional Hazard model using PRS ($P_{Threshold}$=0.005), baseline age, sex, and years of education.

* $P_{Threshold}$=0.005 refers to the p-value threshold used to select SNPs for PRS generation

Table S27: Full model parameter estimates of the time-to-event analysis with the risk-increasing PRS in a ROSMAP APOEε3 homozygote sample

Term	Estimate [95% CI][1]	Std Error[1]	P[1]
Risk-increasing PRS ($P_{Threshold}$=0.005)*	0.006 [0.001, 0.010]	0.002	**0.0101**
Age at Baseline	-0.097 [-0.117, -0.077]	0.010	**2.63e-21**
Sex (female)	-0.123[-0.255, 0.010]	0.068	0.0693
Education	-0.025 [-0.060, 0.010]	0.018	0.1543

[1]Multivariate Cox Proportional Hazard model using risk-increasing PRS ($P_{Threshold}$=0.005), baseline age, sex, and years of education.

* $P_{Threshold}$=0.005 refers to the p-value threshold used to select SNPs for PRS generation

Table S28: Full model parameter estimates of the time-to-event analysis in ROSMAP adjusted with PC1-3

Term	Estimate [95% CI][1]	Std Error[1]	P[1]
Age at Baseline	-0.115 [-0.130, -0.010]	0.008	**1.7e-50**
PRS ($P_{Threshold}$=0.005)*	0.149 [0.065, 0.233]	0.043	**0.001**

Education	-0.045 [-0.072, -0.019]	0.014	**0.001**
Sex (Female)	-0.118 [-0.214, -0.023]	0.049	**0.003**
APOE4[0][2]	-0.308 [-0.504, -0.111]	0.100	**0.017**
APOE4[1][2]	0.182 [-0.223, 0.182]	0.103	**0.017**
PC1	4.14798685 [-3.376, 11.672]	3.839	0.257
PC2	-0.183 [-25.912, 25.547]	13.128	0.651
PC3	-0.929 [-4.970, 3.113]	2.062	0.989

[1]Multivariate Cox Proportional Hazard model using PRS ($P_{Threshold}$=0.005), baseline age, sex, years of education, *APOEε4* allele count, PC1, PC2, and PC3.

[2]Subjects with two *APOEε4* allele copies serve as the reference group.

[*] $P_{Threshold}$=0.005 refers to the p-value threshold used to select SNPs for PRS generation

Table S29: Full model, excluding baseline age, parameter estimates of time-to-event analysis in ROSMAP adjusted with PC1-3

Term	Estimate [95% CI][1]	Std Error[1]	P[1]
PRS ($P_{Threshold}$=0.005)[*]	0.141 [0.058, 0.225]	0.043	**0.001**
Education	0.001 [-0.024, 0.027]	0.013	0.918
Sex (Female)	-0.132 [-0.226, -0.035]	0.049	**0.008**
APOE4[0][2]	-0.427 [-0.612, -0.221]	0.099	**0.0004**
APOE4[1][2]	-0.179 [-0.374, 0.034]	0.103	**0.0004**
PC1	-4.655 [-11.297, 3.307]	3.723	0.169
PC2	-19.690 [-42.119, 9.283]	13.157	0.236
PC3	-1.832 [-5.721, 1.923]	1.950	0.343

[1]Multivariate Cox Proportional Hazard model using PRS ($P_{Threshold}$=0.005), sex, and years of education, *APOEε4* allele count, PC1, PC2, and PC3.

[2]Subjects with two *APOEε4* allele copies serve as the reference group.

[*] $P_{Threshold}$=0.005 refers to the p-value threshold used to select SNPs for PRS generation

Table S30: Full model adjusted with PC1-3 parameter estimates of the time-to-event analysis with the risk-increasing PRS in a ROSMAP sample

Term	Estimate [95% CI][1]	Std Error[1]	P[1]
Age at Baseline	-0.115 [-0.130, -0.100]	0.008	**1.7e-50**
Risk-increasing PRS ($P_{\text{Threshold}}$=0.005)*	0.006 [0.003, 0.009]	0.002	**0.001**
Education	-0.045 [-0.072, -0.019]	0.014	**0.001**
Sex (Female)	-0.118 [-0.214, -0.023]	0.049	**0.017**
APOE4[0][2]	-0.308 [-0.504, -0.111]	0.100	**0.003**
APOE4[1][2]	-0.021 [-0.223, 0.182]	0.103	**0.003**
PC1	4.148 [-3.376, 11.672]	3.839	0.257
PC2	-0.183 [-25.912, 25.547]	13.128	0.989
PC3	-0.929 [-4.970, 3.113]	2.062	0.651

[1]Multivariate Cox Proportional Hazard model using risk-increasing PRS ($P_{\text{Threshold}}$=0.005), baseline age, sex, and years of education, *APOEε4* allele count, PC1, PC2, and PC3.

[2]Subjects with two *APOEε4* allele copies serve as the reference group.

* $P_{\text{Threshold}}$=0.005 refers to the p-value threshold used to select SNPs for PRS generation

Table S31: Full model adjusted with PC1-3 without baseline age parameter estimates of the time-to-event analysis with the risk-increasing PRS in a ROSMAP sample

Term	Estimate [95% CI][1]	Std Error[1]	P[1]
Risk-increasing PRS ($P_{\text{Threshold}}$=0.005)*	0.006 [0.002, 0.009]	0.002	**0.001**
Education	0.001 [-0.024, 0.027]	0.013	**0.027**
Sex (Female)	-0.132 [-0.226, -0.035]	0.049	**0.008**
APOE4[0][2]	-0.427 [-0.612, -0.221]	0.099	**0.0004**

APOE4[1][2]	-0.179 [-0.374, 0.034]	0.103	**0.0004**
PC1	-4.655 [-11.297, 3.307]	3.723	0.236
PC2	-19.690 [-42.119, 9.283]	13.157	0.169
PC3	-1.832 [-5.721, 1.923]	1.950	0.343

[1]Multivariate Cox Proportional Hazard model using risk-increasing PRS ($P_{\text{Threshold}}$=0.005), sex, and years of education, *APOEε4* allele count, PC1, PC2, and PC3.

[2]Subjects with two *APOEε4* allele copies serve as the reference group.

[*] $P_{\text{Threshold}}$=0.005 refers to the p-value threshold used to select SNPs for PRS generation

Table S32: Full model adjusted with PC1-3 parameter estimates of the time-to-event analysis with the PRS in a ROSMAP APOEε3 homozygote sample

Term	Estimate [95% CI][1]	Std Error[1]	P[1]
Age at Baseline	-0.097 [-0.117, -0.077]	0.010	**1.6e-21**
Sex (Female)	-0.122 [-0.253, 0.0129]	0.068	0.076
Education	-0.026 [-0.062, 0.010]	0.018	0.159
PC1	-0.156 [-14.641, 14.486]	7.4301	0.983
PC2	-5.587 [-59.215, 48.187]	27.404	0.838
PC3	-0.990 [-6.700, 4.544]	2.869	0.729
PRS ($P_{\text{Threshold}}$=0.005)[*]	0.137 [0.029, 0.243]	0.055	**0.013**

[1]Multivariate Cox Proportional Hazard model using PRS ($P_{\text{Threshold}}$=0.005), baseline age, sex, years of education, PC1, PC2, and PC3.

[*] $P_{\text{Threshold}}$=0.005 refers to the p-value threshold used to select SNPs for PRS generation

Table S33: Full model adjusted with PC1-3 excluding baseline age parameter estimates of the time-to-event analysis with the PRS in a ROSMAP APOEε3 homozygote sample

Term	Estimate [95% CI][1]	Std Error[1]	P[1]
Sex (Female)	-0.164 [-0.295, -0.030]	0.067	**0.017**
Education	0.022 [-0.014, 0.057]	0.018	0.229

PC1	-7.692 [-21.369, 6.153]	7.021	0.275
PC2	-24.438 [-74.898, 26.215]	25.798	0.344
PC3	-0.969 [-6.282, 4.133]	2.657	0.715
PRS ($P_{Threshold}$=0.005)*	0.115 [0.008, 0.222]	0.055	**0.035**

[1]Multivariate Cox Proportional Hazard model using PRS ($P_{Threshold}$=0.005), sex, years of education, PC1, PC2, and PC3.

* $P_{Threshold}$=0.005 refers to the p-value threshold used to select SNPs for PRS generation

Table S34: Full model adjusted with PC1-3 parameter estimates of the time-to-event analysis with the risk-increasing PRS in a ROSMAP APOEε3 homozygote sample

Term	Estimate [95% CI][1]	Std Error[1]	P[1]
Age at Baseline	-0.097 [-0.117, -0.077]	0.010	**1.6e-21**
Risk-increasing PRS ($P_{Threshold}$=0.005)*	0.005 [0.001, 0.010]	0.002	**0.013**
Education	-0.026 [-0.062, 0.010]	0.018	0.159
Sex (Female)	-0.122 [-0.253, 0.013]	0.068	0.076
PC1	-0.156 [-14.641, 14.486]	7.430	0.983
PC2	-5.587 [-59.215, 48.187]	27.404	0.838
PC3	-0.990 [-6.700, 4.544]	2.869	0.729

[1]Multivariate Cox Proportional Hazard model using risk-increasing PRS ($P_{Threshold}$=0.005), baseline age, sex, years of education, PC1, PC2, and PC3.

* $P_{Threshold}$=0.005 refers to the p-value threshold used to select SNPs for PRS generation

Table S35: Full model adjusted with PC1-3 excluding baseline age parameter estimates of the time-to-event analysis with the risk-increasing PRS in a ROSMAP APOEε3 homozygote sample

Term	Estimate [95% CI][1]	Std Error[1]	P[1]
Risk-increasing PRS ($P_{Threshold}$=0.005)*	0.005 [0.0003, 0.009]	0.002	**0.035**

Education	0.022 [-0.014, 0.057]	0.018	0.229
Sex (Female)	-0.164 [-0.295, -0.030]	0.067	**0.017**
PC1	-7.692 [-21.369, 6.153]	7.021	0.275
PC2	-24.438 [-74.898, 26.215]	25.7986	0.344
PC3	-0.969 [-6.282, 4.133]	2.657	0.714

[1]Multivariate Cox Proportional Hazard model using risk-increasing PRS ($P_{\text{Threshold}}$=0.005), sex, years of education, PC1, PC2, and PC3.

[*] $P_{\text{Threshold}}$=0.005 refers to the p-value threshold used to select SNPs for PRS generation

Table S36: Full model parameter estimates of the time-to-event analysis in NACC

Term	Estimate [95% CI][1]	Std Error[1]	P[1]
Age at Baseline	-0.268 [-0.276, -0.250]	0.004	**4.2e-322**
Education	-0.006 [-0.013, -0.0004]	0.003	**0.033**
Sex (male)	-0.038 [-0.074, -0.001]	0.019	**0.042**
APOEe4[0][2]	-0.020 [-0.077, 0.037]	0.029	0.747
APOEe4[1][2]	0.011 [-0.042, 0.064]	0.027	0.747
PRS ($P_{\text{Threshold}}$=0.005)[*]	0.050 [0.013, 0.087]	0.019	**0.009**

[1]Multivariate Cox Proportional Hazard model using PRS ($P_{\text{Threshold}}$=0.005), baseline age, sex, years of education, and *APOEε4* allele count.

[2]Subjects with two *APOEε4* allele copies serve as the reference group.

[*] $P_{\text{Threshold}}$=0.005 refers to the p-value threshold used to select SNPs for PRS generation

Table S37: Full model parameter estimates of the time-to-event analysis with the risk-increasing PRS in NACC

Term	Estimate [95% CI][1]	Std Error[1]	P[1]
Age at Baseline	-0.268 [-0.276, -0.260]	0.004	**4.2e-322**
Sex (male)	-0.038 [-0.074, 0.001]	0.019	0.043

Education	-0.006 [-0.012, -0.0001]	0.003	**0.045**
Risk-increasing PRS ($P_{Threshold}$=0.005)*	0.0006 [0.0002, 0.001]	0.0002	**0.003**
APOEe4[0][2]	-0.017 [-0.075, 0.040]	0.029	0.788
APOEe4[1][2]	0.012 [-0.042, 0.064]	0.027	0.788

[1]Multivariate Cox Proportional Hazard model using risk-increasing PRS ($P_{Threshold}$=0.005), baseline age, sex, years of education, and *APOEε4* allele count.

[2]Subjects with two *APOEε4* allele copies serve as the reference group.

* $P_{Threshold}$=0.005 refers to the p-value threshold used to select SNPs for PRS generation

Table S38: Full model parameter estimates of the time-to-event analysis in a NACC APOEε3 homozygote sample

Term	Estimate [95% CI][1]	Std Error[1]	P[1]
Age at Baseline	-0.284 [-0.298, -0.270]	0.007	**4.2e-322**
Sex (male)	-0.063 [-0.124, -0.002]	0.031	**0.042**
Education	-0.018 [-0.030, -0.007]	0.006	**0.002**
PRS ($P_{Threshold}$=0.005)*	0.039 [-0.024, 0.103]	0.032	0.227

[1]Multivariate Cox Proportional Hazard model using PRS ($P_{Threshold}$=0.005), baseline age, sex, and years of education.

* $P_{Threshold}$=0.005 refers to the p-value threshold used to select SNPs for PRS generation

Table S39: Full model parameter estimates of the time-to-event analysis with the risk-increasing PRS in a NACC APOEε3 homozygote sample

Term	Estimate [95% CI][1]	Std Error[1]	P[1]
Age at Baseline	-0.284 [-0.298, -0.270]	0.007	**4.2e-322**
Sex (male)	-0.064 [-0.124, -0.003]	0.031	**0.039**
Education	-0.018 [-0.032, -0.008]	0.006	**0.0002**
Risk-increasing PRS ($P_{Threshold}$=0.005)*	-0.0003 [-0.0004, 0.001]	0.0003	0.438

[1]Multivariate Cox Proportional Hazard model using risk-increasing PRS ($P_{Threshold}$=0.005), baseline age, sex, and years of education.

* $P_{Threshold}$=0.005 refers to the p-value threshold used to select SNPs for PRS generation

Table S40: Full model parameter estimates of the time-to-event analysis in NACC adjusted with PC1-3

Term	Estimate [95% CI][1]	Std Error[1]	P[1]
Age at Baseline	-0.268 [-0.277, -0.260]	0.004	**4.2e-322**
PRS ($P_{Threshold}$=0.005)*	0.050 [0.012, 0.088]	0.019	**0.010**
Education	-0.007 [-0.013, -0.0003]	0.003	**0.039**
Sex (male)	-0.038 [-0.075, -0.002]	0.019	**0.040**
APOEe4[0][2]	-0.014 [-0.072, 0.043]	0.029	0.836
APOEe4[1][2]	0.011 [-0.042, 0.064]	0.0277	0.836
PC1	-0.376 [-10.431, 9.678]	5.130	0.942
PC2	2.403 [-2.295, 7.100]	2.397	0.316
PC3	4.473 [-0.691, 9.636]	2.634	0.090

[1]Multivariate Cox Proportional Hazard model using PRS ($P_{Threshold}$=0.005), baseline age, sex, years of education, *APOEε4* allele count, PC1, PC2, and PC3.

[2]Subjects with two *APOEε4* allele copies serve as the reference group.

* $P_{Threshold}$=0.005 refers to the p-value threshold used to select SNPs for PRS generation

Table S41: Full model excluding baseline age parameter estimates of the time-to-event analysis in NACC adjusted with PC1-3

Term	Estimate [95% CI][1]	Std Error[1]	P[1]
Education	0.001 [-0.003, 0.006]	0.002	0.573
Sex (male)	0.034 [-0.002, 0.071]	0.019	0.066
APOEe4[0][2]	-0.455 [-0.511, -0.399]	0.029	**3e-56**

APOEe4[1][2]	-0.014 [-0.066, 0.039]	0.027	**3e-56**
PRS ($P_{Threshold}$=0.005)*	0.011 [-0.027, 0.048]	0.019	0.577
PC1	1.347 [-8.355, 11.048]	4.950	0.786
PC2	-6.959 [-11.586, -2.331]	2.361	**0.003**
PC3	-3.635 [-8.862, 1.592]	2.667	0.173

[1]Multivariate Cox Proportional Hazard model using PRS ($P_{Threshold}$=0.005) sex, years of education, *APOEε4* allele count, PC1, PC2, and PC3.

[2]Subjects with two *APOEε4* allele copies serve as the reference group.

* $P_{Threshold}$=0.005 refers to the p-value threshold used to select SNPs for PRS generation

Table S42: Full model adjusted with PC1-3 parameter estimates of the time-to-event analysis with the risk-increasing PRS in a NACC sample

Term	Estimate [95% CI][1]	Std Error[1]	P[1]
Age at Baseline	-0.269 [-0.277, -0.261]	0.004	**4.2e-322**
Education	-0.007 [-0.013, -0.0004]	0.003	**0.037**
Sex (male)	-0.038 [-0.075, -0.002]	0.019	**0.041**
APOEe4[0][2]	-0.012 [-0.069, 0.046]	0.029	0.870
APOEe4[1][2]	0.010 [-0.043, 0.063]	0.027	0.870
Risk-increasing PRS ($P_{Threshold}$=0.005)*	0.0007 [0.0002, 0.001]	0.0002	**0.002**
PC1	0.317 [-9.712, 10.346]	5.117	0.951
PC2	2.745 [-1.947, 7.437]	2.394	0.252
PC3	4.598 [-0.556, 9.753]	2.630	0.080

[1]Multivariate Cox Proportional Hazard model using risk-increasing PRS ($P_{Threshold}$=0.005), baseline age, sex, years of education, *APOEε4* allele count, PC1, PC2, and PC3.

[2]Subjects with two *APOEε4* allele copies serve as the reference group.

* $P_{Threshold}$=0.005 refers to the p-value threshold used to select SNPs for PRS generation

Table S43: Full model adjusted with PC1-3 excluding baseline age parameter estimates of the time-to-event analysis with the risk-increasing PRS in a NACC sample

Term	Estimate [95% CI][1]	Std Error[1]	P[1]
Education	0.001 [-0.003, 0.006]	0.002	0.604
Sex (male)	0.032 [-0.004, 0.069]	0.019	0.081
APOEe4[0][2]	-0.455 [-0.512, -0.399]	0.029	**8.2e-54**
APOEe4[1][2]	-0.010 [-0.063, 0.042]	0.027	**8.2e-54**
Risk-increasing PRS ($P_{\text{Threshold}}$=0.005)*	-0.0006 [-0.0010, -0.0001]	0.0002	**0.009**
PC1	-1.806 [-11.530, 7.918]	4.961	0.721
PC2	-7.185 [-11.821, -2.548]	2.366	**0.004**
PC3	-4.043 [-9.265, 1.179]	2.664	0.134

[1]Multivariate Cox Proportional Hazard model using risk-increasing PRS ($P_{\text{Threshold}}$=0.005), sex, years of education, *APOEε4* allele count, PC1, PC2, and PC3.

[2]Subjects with two *APOEε4* allele copies serve as the reference group.

* $P_{\text{Threshold}}$=0.005 refers to the p-value threshold used to select SNPs for PRS generation

Table S44: Full model adjusted with PC1-3 parameter estimates of the time-to-event analysis with the PRS in a NACC APOEε3 homozygote sample

Term	Estimate [95% CI][1]	Std Error[1]	P[1]
Age at Baseline	-0.286 [-0.301, -0.272]	0.007	**4.2e-322**
Sex (male)	-0.071 [-0.131, -0.010]	0.031	**0.023**
Education	-0.019 [-0.031, -0.007]	0.006	**0.002**
PRS ($P_{\text{Threshold}}$=0.005)*	0.039 [-0.025, 0.104]	0.033	0.231
PC1	-81.030 [-162.328, 0.268]	41.479	0.051

PC2	-19.946 [-45.965, 6.072]	13.275	0.133
PC3	19.604 [5.047, 34.161]	7.427	0.008

[1]Multivariate Cox Proportional Hazard model using PRS ($P_{\text{Threshold}}$=0.005), baseline age, sex, years of education, PC1, PC2, and PC3.

* $P_{\text{Threshold}}$=0.005 refers to the p-value threshold used to select SNPs for PRS generation

Table S45: Full model adjusted with PC1-3 excluding baseline age parameter estimates of the time-to-event analysis with the PRS in a NACC APOEε3 homozygote sample

Term	Estimate [95% CI][1]	Std Error[1]	P[1]
Sex (male)	0.103 [0.043, 0.163]	0.031	**0.0008**
Education	-0.005 [-0.013, 0.002]	0.004	0.112
PRS ($P_{\text{Threshold}}$=0.005)*	-0.0005 [-0.061, 0.060]	0.031	0.988
PC1	-63.574 [-146.318, 19.168]	42.217	0.126
PC2	-25.065 [-51.366, 1.235]	13.419	0.057
PC3	10.052 [-4.639, 24.743]	7.495	0.175

[1]Multivariate Cox Proportional Hazard model using PRS ($P_{\text{Threshold}}$=0.005), sex, years of education, PC1, PC2, and PC3.

* $P_{\text{Threshold}}$=0.005 refers to the p-value threshold used to select SNPs for PRS generation

Table S46: Full model adjusted with PC1-3 parameter estimates of the time-to-event analysis with the risk-increasing PRS in a NACC APOEε3 homozygote sample

Term	Estimate [95% CI][1]	Std Error[1]	P[1]
Age at Baseline	-0.287 [-0.301, -0.273]	0.007	**4.2e-322**
Sex (male)	-0.071 [-0.132, -0.010]	0.031	**0.022**
Education	-0.019 [-0.033, -0.008]	0.006	**0.0001**

Risk-increasing PRS ($P_{Threshold}$=0.005)*	0.0003 [-0.0004, 0.001]	0.0004	0.332
PC1	-81.483 [-163.843, -1.873]	41.330	**0.045**
PC2	-19.794[-46.075, 5.764]	13.227	0.130
PC3	19.655 [5.355, 34.444]	7.419	**0.007**

[1]Multivariate Cox Proportional Hazard model using risk-increasing PRS ($P_{Threshold}$=0.005), baseline age, sex, years of education, PC1, PC2, and PC3.

* $P_{Threshold}$=0.005 refers to the p-value threshold used to select SNPs for PRS generation

Table S47: Full model adjusted with PC1-3 excluding baseline age parameter estimates of the time-to-event analysis with the risk-increasing PRS in a NACC APOEε3 homozygote sample

Term	Estimate [95% CI][1]	Std Error[1]	P[1]
Sex (male)	0.100 [0.039, 0.150]	0.031	**0.001**
Education	-0.006 [-0.013, 0.001]	0.004	0.098
Risk-increasing PRS ($P_{Threshold}$=0.005)*	-0.0007 [-0.001, 1.02e-5]	0.0004	0.054
PC1	-64.158 [-149.541, 17.839]	42.713	0.127
PC2	-24.420 [-51.503, 1.738]	13.585	0.068
PC3	9.261 [-5.325, 24.361]	7.572	0.216

[1]Multivariate Cox Proportional Hazard model using risk-increasing PRS ($P_{Threshold}$=0.005), sex, years of education, PC1, PC2, and PC3.

* $P_{Threshold}$=0.005 refers to the p-value threshold used to select SNPs for PRS generation

Supplementary References

1. DeLong ER, DeLong DM, Clarke-Pearson DL. Comparing the areas under two or more correlated receiver operating characteristic curves: a nonparametric approach. Biometrics. 1988;44(3):837-45.
